# Assessing the efficacy, safety and utility of fully closed-loop insulin delivery compared to standard insulin therapy with a continuous glucose monitor in adults with type 2 diabetes (COYOTE study): a randomised parallel study protocol

**DOI:** 10.1136/bmjopen-2025-115464

**Published:** 2026-05-19

**Authors:** R Seese, CK Boughton, FT Tseung, R Uy, ME Wilinska, H Thabit, YS Cheah, S Neupane, S Hussain, P Choudhary, EG Wilmot, L Bally, H Hanaire, JK Mader, M Haluzík, D O’Neal, J Lawton, D Rankin, C Kollman, G Dunseath, R Hovorka

**Affiliations:** 1 Institute of Metabolic Science-Metabolic Research Laboratories, University of Cambridge, Cambridge, England, UK; 2 Wolfson Diabetes and Endocrine Clinic, Cambridge University Hospitals NHS Foundation Trust, Cambridge, UK; 3 Manchester Royal Infirmary, Central Manchester University Hospitals NHS Foundation Trust, Manchester, UK; 4 Faculty of Biology, Medicine and Health, The University of Manchester, Manchester, UK; 5 King’s College Hospital, King’s College Hospital NHS Foundation Trust, London, UK; 6 Norwich Medicine School, University of East Anglia, Norwich, UK; 7 Norfolk and Norwich University Hospital, Norfolk and Norwich University Hospitals NHS Foundation Trust, Norwich, UK; 8 Department of Diabetes, School of Cardiovascular, Metabolic Medicine and Sciences, King’s College London, London, UK; 9 Department of Diabetes and Endocrinology, Guy’s & St Thomas’ NHS Foundation Trust, London, UK; 10 Institute of Diabetes, Endocrinology and Obesity, King’s Health Partners, London, UK; 11 Leicester Diabetes Centre, University Hospitals of Leicester NHS Trust, Leicester, UK; 12 School of Medicine, University of Nottingham, Nottingham, UK; 13 University Hospitals of Derby and Burton NHS Foundation Trust, Derby, UK; 14 Inselspital University Hospital Bern, Bern, Switzerland; 15 University of Toulouse, Toulouse, France; 16 Medical University of Graz, Graz, Austria; 17 Institute for Clinical and Experimental Medicine, Prague, Czech Republic; 18 Department of Medicine St. Vincent’s Hospital Melbourne, The University of Melbourne, Melbourne, Victoria, Australia; 19 Baker Heart and Diabetes Institute, Melbourne, Victoria, Australia; 20 School of Population Health Sciences, The University of Edinburgh Usher Institute, Edinburgh, UK; 21 The Jaeb Center for Health Research, Tampa, Florida, USA; 22 Diabetes Research Group, Swansea University, Swansea, UK

**Keywords:** Diabetes Mellitus, Type 2, Clinical Protocols, DIABETES & ENDOCRINOLOGY

## Abstract

**Introduction:**

Type 2 diabetes (T2D) presents a global healthcare burden. Despite widespread use of non-insulin glucose lowering therapies, many individuals still require insulin to achieve recommended target glycated haemoglobin (HbA1c). Insulin injections improve HbA1c but can lead to problematic hypoglycaemia. The objective of this study is to determine whether fully closed-loop insulin delivery improves HbA1c at 26 weeks compared with standard insulin therapy with continuous glucose monitoring (CGM) in adults with T2D.

**Methods and analysis:**

This study adopts an open-label, multinational, randomised, single-period parallel design and aims to randomise 224 adults with T2D to either standard insulin therapy with CGM (control group) or fully closed-loop insulin delivery (intervention group) for a period of 26 weeks. Participants will complete a run-in period of 2–3 weeks wearing a masked CGM followed by randomisation to either the control or intervention group. The primary endpoint is the between-group difference in HbA1c at 26 weeks. Key endpoints include time in target glucose range (3.9–10.0 mmol/L), mean sensor glucose, time above range (>10.0 mmol/L) and non-inferiority for time below target (<3.9 mmol/L) over the 26-week study period. Secondary outcomes include standard CGM metrics, binary metrics for HbA1c, total daily insulin dose, body mass index, blood pressure, fasted lipid profile, renal function and liver function. Safety will be assessed by the frequency of severe hypoglycaemic episodes and other adverse events. Utility will be assessed by CGM and closed-loop system use. The impact of fully closed-loop will be assessed using validated questionnaires and interviews.

**Ethics and dissemination:**

The study has received ethical approval from the East of England Cambridgeshire and Hertfordshire Research Ethics Committee (24/EE/0149) in the UK. Ethical approval for non-UK site has been obtained by local Research Ethics Committees.

Results will be disseminated by peer-reviewed publications and conference presentations, and findings will be shared with people living with diabetes, healthcare providers and relevant stakeholders.

**Trial registration number:**

NCT06579404.

**STRENGTHS AND LIMITATIONS OF THIS STUDY**

- The study adopts an open-label, multinational, randomised parallel design and includes a 26-week follow-up period with equal numbers of study visits between both groups.
- The comparator therapy includes standard insulin injections with continuous glucose monitoring, reflecting the most advanced diabetes technology used in current standard of care.
- The study has broad inclusion criteria and limited exclusion criteria to support generalisability of the findings.
- Exclusion of pregnant participants or participants planning pregnancy limits generalisability.
- Randomisation is not stratified by insulin or non-insulin glucose lowering therapies. The proportion of participants using basal or more intensive insulin therapy may differ between groups.

## Introduction

 Type 2 diabetes (T2D) poses a significant healthcare burden across the world with an estimated 11.1% of adults living with the condition worldwide with prevalence projected to rise.^1^ The proportion of T2D in younger people is rising, with an estimated 168 000 people with T2D under the age of 40 in the UK alone.^2^ Complications of suboptimal management of T2D include microvascular and macrovascular disease and prevention of these remains the primary aim of treatment. The ADVANCE trial showed reduction in risk of microvascular disease when achieving a target HbA1c <53 mmol/mol (7%),^3^ however the ACCORD trial revealed an increased risk of mortality and no reduction in cardiovascular events with a lower HbA1c target <42 mmol/mol (6%), postulated to be a consequence of an increase in hypoglycaemic events.^4^

In an attempt to achieve the National Institute for Health and Care Excellence guidance target of HbA1C of <53 mmol/mol (7%), one in four people with T2D require insulin despite the increasing use of oral and injectable non-insulin therapies.^5^ However, only 50% of people with T2D reach target HbA1c.^6^ Limitations of multiple daily injections include hypoglycaemia and weight gain, which can contribute towards deterioration of cardiovascular risk factors such as obesity and glycaemic outcomes. Delivery of insulin, for those who need it, using a fully automated closed-loop system (no meal announcements) provides a realistic opportunity to tailor insulin delivery to better achieve glycaemic targets and reduce the risk of complications and subsequent burden for the person with diabetes and healthcare providers.

We have previously shown in a small (n=26) single centre-study that fully closed-loop insulin therapy in comparison to standard insulin therapy can significantly improve HbA1c without increasing the risk of hypoglycaemia in adults with T2D.^7^ We aim to determine whether these benefits are reproducible in a larger, more diverse population with T2D and hypothesise that fully closed-loop delivery will be safe and improve glycaemic outcomes compared with standard insulin therapy with CGM. We will also explore the experiences of system use from participants’ and healthcare professionals’ perspectives.^8^

## Methods and analysis

### Overview

**Figure 1:**
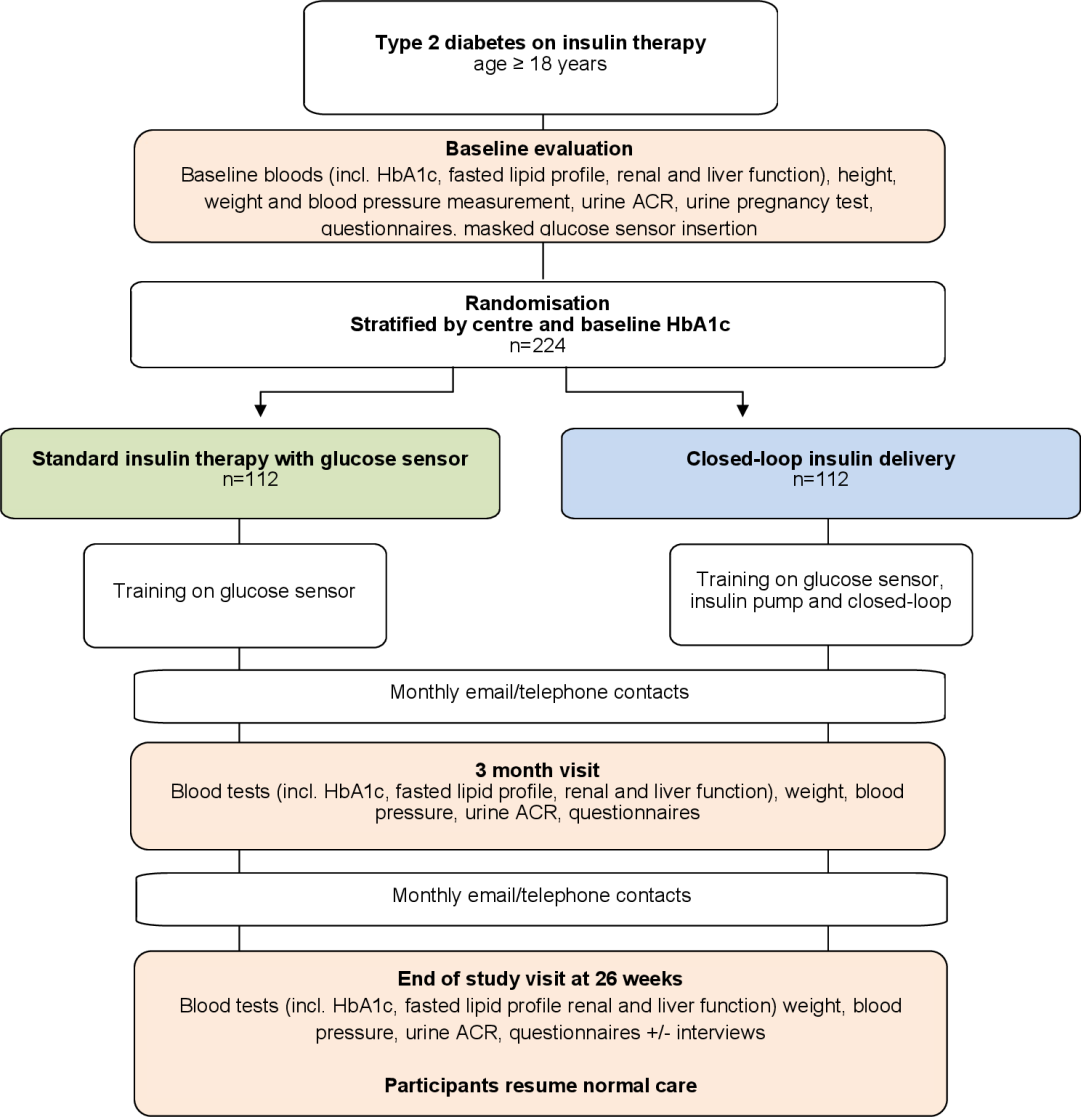
Study flow chart. ACR, albumin creatinine ratio; HbA1c, glycated haemoglobin.

This study adopts an open-label, multinational, multicentre, randomised, single-period parallel design to assess the efficacy, safety and utility of fully closed-loop insulin delivery compared with standard insulin therapy with CGM in adults with T2D over 26 weeks (figure 1). The full study protocol can be found in the supplementary material. Participants with T2D ≥12 months must be established on insulin therapy for 6 months or more. In addition, participants must be established on a sodium-glucose co-transporter-2 (SGLT2) inhibitor and/or a Glucagon-like peptide-1 (GLP-1) receptor agonist for 3 months or have been offered this therapy to reflect current standard of care. We will aim for 25% of participants to be using basal insulin only and 60% basal bolus insulin therapy at baseline. This study aims to randomise 244 participants (122 per group). The primary endpoint is the between group difference in centralised measurement of HbA1c at 26 weeks. The CamAPS HX fully closed-loop system will be used in the intervention arm, comprising Ypsomed insulin pump (Mylife Diabetes Care, Burgdorf, Switzerland), FreeStyle Libre 3 CGM sensor (Abbott, Illinois, USA) and a compatible smartphone to host the CamAPS HX app (CamDiab, Cambridge, UK).

### Clinical sites

The University of Cambridge, UK will be the coordinating centre. Clinical trial sites include:

1. Addenbrooke’s Hospital, Cambridge University Hospitals NHS Foundation Trust, UK.
2. University Hospitals of Derby and Burton NHS Foundation Trust, Derby, UK.
3. Manchester Royal Infirmary, Manchester University NHS Foundation Trust, UK.
4. King’s College Hospital, King’s College Hospital NHS Foundation Trust, London, UK.
5. Guy’s and St Thomas’ NHS Foundation Trust, London, UK.
6. Norfolk and Norwich University Hospital, Norfolk and Norwich University Hospitals NHS Foundation Trust, UK.
7. University Hospitals of Leicester NHS Trust, Leicester, UK.
8. Inselspital, Bern University Hospital, Bern, Switzerland.
9. Centre Hospitalier Universitaire de Toulouse, France.
10. Medical University of Graz, Graz, Austria.
11. Diabetes Centre, Institute for Clinical and Experimental Medicine, Prague, Czech Republic.
12. St Vincent’s Hospital, Melbourne, Australia.

Participants will be recruited through outpatient diabetes clinics, primary care centres, social media advertising or other established methods at participating centres. General practice surgeries in the region may be included as participant identification centres for the UK study sites. Each centre will aim to recruit between 15–25 participants.

The maximum duration of study for a participant will be 30 weeks.

### Inclusion criteria

1. Aged 18 years and older.
2. T2D diagnosed for at least 12 months.
3. Established on an SGLT2 inhibitor and/or GLP-1 receptor agonist for at least 3 months or have been offered these therapies previously.
4. Treatment with insulin therapy for at least 6 months.
5. HbA1c ≤140 mmol/mol (≤15%) analysis from local laboratory or equivalent.
6. Willing to wear study devices and follow study instructions.
7. Capacity to consent to participate in the study.

### Exclusion criteria

1. Type 1 diabetes.
2. Current use of insulin pump.
3. Current use of any closed-loop system.
4. Any physical/psychological disease or medication(s) likely to interfere with the conduct of the study and interpretation of the study results, as judged by study clinician.
5. Known or suspected allergy against insulin.
6. Pregnancy, planned pregnancy or breast-feeding.
7. Severe visual impairment.
8. Severe hearing impairment.
9. Medically documented allergy towards the adhesive (glue) of plasters.
10. Serious skin diseases located at places of the body which are potential areas used for localisation of the glucose sensor.
11. Illicit drug abuse.
12. Prescription drug abuse.
13. Alcohol abuse.

Potential participants with diabetes-related retinal disease will not be excluded from the trial. An individualised risk approach will be used, and we will consider delaying recruiting people undergoing active treatment of proliferative retinopathy/maculopathy until treatment is complete.

### Study schedule

#### Overview

Written informed consent will be obtained from all participants before any study-related activities. Participants will have a run-in period of 2–3 weeks where they wear a masked CGM and continue with their usual diabetes management. To proceed with the study, at least 10 days of CGM data will be required during the run-in period. Participants will then be randomised to 26 weeks of either fully closed-loop insulin therapy or standard insulin therapy with CGM. The study comprises up to five visits and six telephone/email contacts (tables1  2). Once randomised, participants will receive contact after 48 hours, 1 week and monthly thereafter until completion of the study. Participants will continue with their regular clinical care alongside study participation. A subset of participants randomised to fully closed-loop will participate in an interview sub-study after at least 3 months of experience using the technology. At the conclusion of the study, participants will resume their standard diabetes management and return the study devices.

**Table 1:** Schedule of study visits/contacts when the participant is randomised to closed-loop (intervention group)

	Visit/contact	Description	Start relative to previous/next visit/activity	Duration
Baseline and run-in	**Visit 1**	Recruitment visit: consent, baseline bloods (including HbA1c, fasted lipid profile, renal and liver function) height, weight and BP, urine ACR, urine pregnancy test, questionnaires, masked CGM insertion		1–2 hours
	**Visit 2**	Review of baseline bloods and CGM data. Randomisation	2–3 weeks after visit 1 (±1 week);	30 min
Postrandomisation training	**Visit 3**	Study pump, CGM and closed-loop training and initiation, competency assessment	may coincide with visit 2, within 2–4 weeks of visit 1	1–2 hours
Closed loop intervention (6 months)	Contact 1	Review use of study devices; study update	within 48 hours after visit 3	30 min
	Contact 2	Review use of study devices; study update	1 week after randomisation (±3 days)	30 min
	Contact 3	Review use of study devices; study update	1 month after randomisation (±2 weeks)	30 min
	Contact 4	Review use of study devices; study update	2 months after randomisation (±2 weeks)	30 min
	**Visit 4**	3-month visit, HbA1c, fasted lipid profile, renal and liver function, weight, BP, urine ACR, questionnaires	3 months after randomisation (±2 weeks)	1 hour
	Contact 5	Review use of study devices; study update	4 months after randomisation (±2 weeks)	30 min
	Contact 6	Review use of study devices; study update	5 months after randomisation (±2 weeks)	30 min
	**Visit 5**	End of closed-loop treatment arm; bloods (HbA1c, fasted lipid profile, renal and liver function), weight, BP, urine ACR, questionnaires ± interviews; resume usual care	6 months after randomisation (±2 weeks)	1–2 hours

Bold is the visits while non-bold is contacts.

ACR, albumin creatinine ratio; BP, blood pressure; CGM, continuous glucose monitoring; HbA1c, glycated haemoglobin.

**Table 2:** Schedule of study visits/contacts when the participant is randomised to standard insulin therapy + CGM (control group)

	Visit/ contact	Description	Start relative to previous/next visit/activity	Duration
Baseline and run-in	**Visit 1**	Recruitment visit: consent, baseline bloods (including HbA1c, fasted lipid profile, renal and liver function), height, weight and BP, urine ACR, urine pregnancy test, questionnaires, masked CGM insertion		1–2 hours
	**Visit 2**	Review of baseline bloods and CGM data. Randomisation	2–3 weeks after visit 1 (±1 week);	30 min
Postrandomisation training	**Visit 3**	CGM training and initiation, competency assessment	May coincide with visit 2, within 2–4 weeks of visit 1	1–2 hours
Standard insulin+CGM (control) (6 months)	Contact 1	Review use of study devices; study update	within 48 hours after visit 3	30 min
	Contact 2	Review use of study devices; study update	1 week after randomisation (±3 days)	30 min
	Contact 3	Review use of study devices; study update	1 month after randomisation (±2 weeks)	30 min
	Contact 4	Review use of study devices; study update	2 months after randomisation (±2 weeks)	30 min
	**Visit 4**	3-month visit, HbA1c, fasted lipid profile, renal and liver function, weight, BP, urine ACR, questionnaires	3 months after randomisation (±2 weeks)	1 hour
	Contact 5	Review use of study devices; study update	4 months after randomisation (±2 weeks)	30 min
	Contact 6	Review use of study devices; study update	5 months after randomisation (±2 weeks)	30 min
	**Visit 5**	End of standard insulin + CGM treatment arm; bloods (HbA1c, fasted lipid profile, renal and liver function), weight, BP, urine ACR, questionnaires ± interviews; resume usual care	6 months after randomisation (±2 weeks)	1–2 hours

ACR, albumin creatinine ratio; BP, blood pressure; CGM, continuous glucose monitoring; HbA1c, glycated haemoglobin.

### Baseline and run-in

#### Recruitment

Potential participants will be given the study information leaflet and invited to join the study by the research team with sufficient time to consider the information before the recruitment visit.

### Prerandomisation

#### Visit 1

Participants will be given a verbal explanation of the study design and purpose. They will be provided the opportunity to ask questions and can request more time to consider participation if desired. Written informed consent will be obtained following confirmation that inclusion/exclusion criteria apply before any study-related activities.

The baseline visit will be used to collect data about demographics, medical and diabetes history, concomitant medications and insulin therapy. Blood tests for HbA1c, fasted lipid profile, renal and liver function, urine albumin creatinine ratio (ACR) and urine pregnancy test for all females aged between 18 years and 60 years will be collected. Height, weight, waist hip ratio and blood pressure (BP)will be measured. A masked CGM will then be applied, and participants will be asked to complete quality of life and diabetes management questionnaires.

Participants will continue with their usual insulin management during the run-in period of 2–3 weeks. If they already use a CGM, they can continue to do so and will be asked to wear two devices (one masked plus their regular) for the duration of the run-in period.

#### Visit 2 (randomisation)

At the end of the run-in period baseline HbA1c and CGM data will be reviewed. To proceed to randomisation, at least 10 days of CGM data are required. Eligible participants will be randomised in a 1:1 ratio using a centrally administered web-based randomisation sequence with a permuted block design (block sizes two and four) to the use of fully closed-loop or standard insulin therapy with CGM for 26 weeks. The randomisation will be stratified by site and baseline HbA1c (≤8.5% (69 mmol/mol)/>8.5% (69 mmol/mol)).

### Postrandomisation training

#### Visit 3

##### Fully closed-loop therapy (intervention)

Participants will receive training from a member of the study team on the use of the study insulin pump, study CGM and CamAPS HX App. Written guidelines will also be provided. Competency will be assessed, and only if participants are deemed competent, will they be able to progress to the home phase of the study. Further training may be delivered as required.

Participants will use the closed-loop system for the next 26 weeks at home.

##### Standard insulin therapy with CGM (control)

Participants will receive training from a member of the study team on the use of the study CGM. Competency will be assessed and only if participants are deemed competent, will they be able to progress to the home phase of the study. Further training may be delivered as required.

Participants will use the CGM system for the next 26 weeks at home.

For both study groups, non-insulin diabetes therapies will be continued, and their usual diabetes care will continue alongside study participation. Participants and/or the clinical team are free to adjust diabetes therapy as per usual clinical practice. Participants are advised to contact the study team if any problems occur with the study devices.

### Contacts after initiation of treatment arm (control and intervention group)

Participants will be contacted by telephone or email at 48 hours and at 1 week after visit 3. The purpose of this contact would be to troubleshoot any issues with the devices, record any adverse events, device deficiencies as well as changes in medical conditions and/or medication.

Thereafter, participants will be followed up through study contacts (telephone/email) at monthly intervals to record any adverse events, device deficiencies, changes in insulin doses, other medical conditions and/or medication.

#### Visit 4 (3-month visit)

Participants will attend for visit 4 approximately 13 weeks after visit 3. Participants will have a blood test for HbA1c, fasted lipid profile, renal function and liver function. Participants’ weight, waist hip ratio and BP will be recorded and urine ACR measured. Participants will be asked to complete questionnaires to assess quality of life and diabetes management.

#### Visit 5 (6-month visit/end of study)

The participant will attend their research centre approximately 26 weeks after visit 3. This will be the end of study visit. Participants will have a blood test for HbA1c, fasted lipid profile, renal function and liver function. Participant’s weight, waist hip ratio and BP will be recorded and urine ACR measured. Participants will be asked to complete questionnaires to assess quality of life and diabetes management. Study devices will be collected, and participants will resume their usual diabetes care. A subset of intervention arm participants will be invited to take part in a qualitative interview.

### Participant withdrawal criteria

#### The following prerandomisation withdrawal criterion will apply

1. Participant is unable to demonstrate safe application of insulin therapy as judged by the investigator.

#### The following prerandomisation and postrandomisation withdrawal criteria will apply

1. Participant is unable to demonstrate safe use of insulin injections or study insulin pump, CGM and/or closed-loop during post randomisation training period as judged by the investigator.
2. Participants may terminate participation in the study at any time without necessarily giving a reason and without any personal disadvantage.
3. Significant protocol violation or non-compliance.
4. Recurrent severe hypoglycaemia events related to the use of the closed-loop system.
5. Recurrent severe persistent hyperglycaemia events/diabetic ketoacidosis (DKA) unrelated to infusion site failure and related to use of the closed-loop system.
6. Decision by the investigator or the Sponsor that termination is in the participant’s best medical interest.
7. Allergic reaction to insulin or severe reaction to adhesive surface of infusion set or glucose sensor.

### Study procedures

#### Height, weight, waist hip ratio and blood pressure

Height will be measured at the baseline visit in centimetres using calibrated measuring devices. Weight will be measured at the baseline visit, 3 month visit and end of study visit in kilograms using a calibrated electronic scale. Waist hip ratio will be measured at the baseline visit, 3-month visit and end of study visit in centimetres using a stretch-resistant tape. Blood pressure will be measured at the baseline visit, 3-month visit and end of study visit in mm Hg, using an approved BP monitor.

#### Venepuncture

Venous blood samples for the measurement of HbA1c levels will be taken at baseline, at 3 months and at the final study visit. HbA1c will be measured at a central laboratory (Swansea University) using an International Federation of Clinical Chemistry and Laboratory Medicine and National Glycohemoglobin Standardization Program certified HPLC method (Tosoh GX).

Venous blood samples for the measurement of fasted lipid profile, renal function (sodium, potassium, urea, serum creatinine, estimated glomerular filtration rate) and liver function (alanine transaminase, aspartate transaminase, alkaline phosphatase, gamma-glutamyltransferase, bilirubin, albumin and fibrosis-4 index) will be taken at baseline, at 3 months and at the final study visit and will be analysed locally. Blood samples will be disposed of after analysis.

#### Urine albumin creatinine ratio

A urine sample for the measurement of urine ACR will be taken at the baseline visit, 3-month visit and end of study visit and analysed locally. Urine samples will be disposed of after analysis.

#### Masked CGM

During the run-in period, participants will wear a masked Libre V.3 sensor to collect baseline CGM data and to determine eligibility for randomisation.

### Human factor assessments

#### Questionnaires

Surveys used in this trial are listed in table 3. Surveys will be completed at the baseline visit, at the 3-month visit and at the end of study visit. Responses will be evaluated at the end of the study once all participants have completed the final study visit.

**Table 3:** Human factors assessment

Measure	Construct measured/relevant points
Surveys	
Problem Areas in Diabetes Survey	20-item survey measuring diabetes-related emotional distress and covers a range of negative emotional problems of patients with diabetes.
Hypoglycaemia Fear Survey-version II	33-item questionnaire with two subscales that measure (1) behaviours to avoid hypoglycaemia and its negative consequences and (2) worries about hypoglycaemia and its negative consequences (5 min).
European Quality of Life 5 Dimensions 3 Level Version (EQ-5D-3L)	Developed to describe and value health across a wide range of disease areas. The survey consists of two pages: the EQ-5D descriptive system assessing five dimensions of health and the EQ-5D visual analogue scale.
WHO-5 quality of life measure	Five-item measure from the WHO that assesses health related quality of life.
Closed-loop experience questionnaire	Feedback questionnaire on closed-loop specific experience will be completed by participants been randomised to the closed-loop intervention arm.

#### Participant interviews (UK only)

The qualitative evaluation will use a cross-sectional design in which approximately n=30 participants in the closed-loop arm at UK sites will be interviewed after they have ≥3 months experience using the system. Purposive sampling will be used to ensure diversity in terms of age, gender and T2D duration, with a particular focus on recruiting those from ethnic minority and/or lower socioeconomic backgrounds. Interviews will explore participants’ pre-trial diabetes management practices, everyday lives and initial expectations of using fully closed-loop technology before considering their views about how using the system has affected their diabetes self-management and everyday lives. Interviews will also explore participants’ views about the training, information and support needed to gain optimal benefit from using the system in routine clinical care.

#### Healthcare professional interviews (UK only)

We will invite approximately 20 healthcare professionals who are involved in supporting people using closed-loop during the trial to take part in an interview at or near the end of their involvement in the trial. Interviews will explore their experiences of supporting people with T2D using a fully closed-loop system during the trial and their views about the training and resourcing needed to support system use in routine clinical care.

### Patient and public involvement

Input on the study design and endpoints was received from previous study participants with lived experience of T2D. To develop our qualitative research question, we involved people living with T2D using insulin from minority ethnic and/or lower socioeconomic backgrounds. These individuals supported the inclusion of people from diverse ethnic and socioeconomic backgrounds to better understand the experiences of, and ensure findings were relevant to, seldom-heard groups using closed-loop technology. Our patient and public involvement (PPI) advisory group will provide ongoing input during the lifetime of the study and support with dissemination of the findings.

### Statistical analysis

All randomised participants with/without protocol violation and including dropouts and withdrawals will be included in the analysis according to intention-to-treat principle.

### Primary endpoint analysis

The primary analysis will evaluate the between-group difference in HbA1c at the end of the 26-week intervention period.

Mean±SD or summary statistics appropriate to the distribution will be reported for the primary endpoint by treatment intervention. The treatment interventions will be compared using a linear mixed model with the dependent variable being the primary endpoint and the independent variable being treatment allocation adjusting for baseline HbA1c. A 95% CI will be reported for the difference between the interventions based on the linear mixed model. Residual values will be examined for an approximate normal distribution. If values are highly skewed, then a transformation or robust statistical method will be used instead.

A 5% significance level will be used to declare statistical significance for the primary comparison. A two-sided p value will be reported. The primary analysis will be a single statistical comparison of a single outcome measure.

A centre-effect will be explored in the analyses by evaluating for interaction between centre and treatment group on the primary outcome.

### Key and secondary endpoint analysis

Analysis of secondary endpoints will parallel the primary analysis. A transformation will be applied to all highly skewed secondary endpoints. The following key and secondary endpoints will be assessed for between-group difference:

#### Key endpoints (throughout treatment period)

- Proportion of time spent in the target glucose range (3.9–10.0 mmol/L).
- Mean sensor glucose.
- Proportion of time spent above target glucose (>10.0 mmol/L).
- Non-inferiority for time spent below target glucose (<3.9 mmol/L).

#### Secondary glucose endpoints (throughout treatment period)

- SD (mmol/L) and coefficient of variation (%) of glucose,
- Proportion of time with glucose <3.5 mmol/L and <3.0 mmol/L.
- Proportion of time with glucose >13.9 mmol/L, >16.7 mmol/L and >20.0 mmol/L.
- Binary metrics for HbA1c (HbA1c <53 mmol/L (7.0%), HbA1c <58 mmol/mol (7.5%)).

Trends in CGM and insulin data collected within intervention arms will be evaluated monthly and daytime (0600–2359) and overnight (0000–0559) glucose control will be evaluated separately.

#### Insulin and other clinical endpoints

- Total daily insulin dose (units/day)
- Body weight (kg) and body mass index (kg/m^2^).
- Waist hip ratio.
- Blood pressure (mm Hg).
- Fasted lipids.
- Renal function as measured by serum creatinine, estimated glomerular filtration rate and urinary ACR.
- Liver function as measured by liver markers (ALT, AST, ALP, yGT, bilirubin and albumin) and FIB4 score.

The Hochberg method will be used to adjust for multiple testing of secondary endpoints.

### Safety analysis

For each of the following safety outcomes, mean±SD or summary statistics appropriate to the distribution will be tabulated by treatment group:

Events will be recorded for all participants including dropouts and withdrawals, regardless of whether CGM data are available and irrespective of whether closed-loop was operational.

- Number of severe hypoglycaemia events.
- Number of participants with any severe hypoglycaemia event.
- Number of diabetic ketoacidosis events.
- Number of participants with any diabetic ketoacidosis event.
- Number of adverse events per participant.
- Number of serious adverse events per participant.

All serious adverse events will be listed for the entire study duration. For severe hypoglycaemia (if enough events), the event rates will be compared using a repeated measures regression model.

### Utility evaluation

The amount of CGM use will be tabulated for each treatment arm, in addition to the amount of closed-loop system use in the closed-loop arm. Summary statistics appropriate to the distribution and range will be reported.

### Questionnaires: analysis

Descriptive tabulations of questionnaires will be carried out, and scores will be calculated using provided scaling and scoring tools as appropriate. The between-group difference of each score will be assessed using a linear mixed model, adjusting for the corresponding score at baseline.

### Participant and healthcare professional interviews: analysis

Data will be analysed thematically, using the method of constant comparison and inductive and deductive approaches. NVivo, a qualitative data indexing software package, will be used to facilitate data coding and retrieval.

### Per-protocol analysis

A per-protocol analysis restricted to participants with a minimum of 60% CGM data during the control period and 60% use of closed-loop during the closed-loop period will be conducted for the primary endpoint.

### Interim analysis

No formal interim analysis will be performed.

### Power calculation

A sample size of 96 participants (48 per group) was determined to have 90% power to detect a difference in mean HbA1c level between treatment groups, assuming a population difference of 0.8%, an effective SD of the 26-week values of 1.2, and a two-sided type 1 error rate of 0.05. This number was increased to 224 (112 per group) to account for dropouts (assuming a 20% dropout rate) and to increase the number of participants who will be exposed to the closed-loop system for an enhanced safety and feasibility assessment. A treatment group difference of ≥0.5% represents a clinically meaningful change in the HbA1c distribution and is generally associated with at least a 50% greater proportion of participants experiencing improvement in HbA1c level by 10% or more, which was shown in the Diabetes Control and Complications Trial to be associated with a substantial risk reduction in diabetic microvascular complications.^9^

### Study management

#### Study sponsor

The study sponsor for UK sites is Cambridge University Hospitals NHS Foundation Trust, jointly with University of Cambridge. Each site outside of the UK is its own sponsor (table 4).

**Table 4:** Investigation site-specific sponsor

Investigation site	Sponsor
UK	Cambridge University Hospitals NHS Foundation Trust, jointly with University of Cambridge
Switzerland	Inselspital, Bern University Hospital University of Bern
France	University Hospital Toulouse, France
Australia	St Vincent’s Hospital Melbourne Research Governance Unit
Czech Republic	Diabetes Centre, Institute of Clinical and Experimental Medicine, Vídeňská
Austria	Medical University of Graz

### Data management overview

An independent data safety monitoring board (DSMB) will oversee the study, safeguarding the interests of trial participants, assess the safety data during the trial and monitor the overall conduct of the clinical trial. The DSMB will review the progress and accruing data of the clinical trial and provide advice on the conduct of the trial. The DSMB will be informed of any related serious adverse events and any unanticipated adverse device effects that occur during the study and will review compiled adverse event data at periodic intervals.

### Trial management group

The trial management group (TMG) will meet weekly and will be responsible for day-to-day management of the trial. The TMG will consist of the chief investigator and study coordinator.

### Data management and monitoring

The study coordinator and independent monitor will be responsible for maintaining quality assurance and quality control systems to ensure that the trial is conducted and data are generated, documented and reported in compliance with the protocol, Good Clinical Practice and Ethics requirements.

Confidentiality of participant data shall be observed at all times. Personal details for each participant with a link to a unique identification number will be held locally in the Trial Site File at each study site. Electronic case report forms will be used for recording anonymised study data and will be completed in accordance with GCP and ISO 14155:2020 Guidelines.

### Indemnity

The clinical investigators are indemnified to cover negligent harm to patients participating in the study by their membership of medical defence organisations. Site specific indemnity cover will apply for any claims arising from management and conduct of research. Any liability arising from study design will be covered by the Sponsor’s clinical trial insurance policy.

## Ethics and dissemination

Ethics approval for the study has been received for each sponsor organisation before commencement of the study (table 5). Studies will be run in accordance with Good Clinical Practice and ethical principles that have their origin in the Declaration of Helsinki. All participants will be provided with verbal and written information about the trial and procedures before obtaining written informed consent.

**Table 5:** Site-specific research ethics committee

Investigation site	Research ethics committee
UK	East of England—Cambridgeshire and Hertfordshire Research Ethics Committee
Switzerland	Cantonal Ethics Committee Bern
France	Northwest II Committee for the Protection of Persons
Australia	St Vincent’s Hospital Melbourne Human Research Ethics Committee
Prague	Ethics Committee of the Institute for Clinical and Experimental Medicine and the Thomayer University Hospital
Austria	Ethics Committee of the Medical University of Graz

Standard operating procedures for monitoring and reporting of all adverse events and adverse device effects will be in place including serious adverse events, serious adverse device effects and specific adverse events such as severe hypoglycaemia and significant hyperglycaemia.

Any substantial amendments to the protocol and other documents shall be notified to and approved by the Research Ethics Committees prior to implementation as per nationally agreed guidelines.

Screening and recruitment commenced in December 2024, and the study is expected to be completed by June 2027. Study results will be disseminated through peer-reviewed publications, conference presentations and lay communications.

### Expected impact

Recent randomised controlled trial evidence shows that hybrid closed-loop insulin delivery is safe and improves glucose outcomes in people with T2D using insulin.^10 11^ Fully automated closed-loop insulin delivery does not require user-initiated insulin delivery and therefore reduces user burden and eliminates the need for healthcare professional optimisation after initial training on the devices. CamAPS HX fully closed-loop system has been shown to improve glucose levels in small randomised controlled trials in adults with T2D requiring insulin.^7^ This large, multinational study aims to investigate whether these benefits of fully closed-loop insulin delivery are observed in a larger and more diverse population of adults with T2D and provide evidence to support reimbursement and adoption.

## Supplementary material

10.1136/bmjopen-2025-115464 online supplemental file 1
