# Supplementary material for: Assessing the efficacy, safety and utility of fully closed-loop insulin delivery compared to standard insulin therapy with a continuous glucose monitor in adults with type 2 diabetes (COYOTE study): a randomised parallel study protocol
Source: BMJ Open. 2026 May 19;16(5):e115464. doi: 10.1136/bmjopen-2025-115464 (PMC13202074; doi:10.1136/bmjopen-2025-115464)
Supplement: online supplemental file 1 [file bmjopen-16-5-s001.pdf]

UNIVERSITY OF CAMBRIDGE

NHS Cambridge University Hospitals NHS Foundation Trust

Clinical Study Protocol

Study Title: An open-label, multi-national, multi-centre, randomised, single-period parallel study to assess the efficacy, safety and utility of fully closed-loop insulin delivery compared to standard insulin therapy with CGM in adults with type 2 diabetes (COYOTE study).

Short Title: Closed-loop in adults with type 2 diabetes

Protocol Version: 6.0 14 October 2025

**Coordinating Investigator**	Prof Roman Hovorka<br>University of Cambridge Metabolic Research Laboratories<br>Level 4, Wellcome-MRC Institute of Metabolic Science<br>Box 289, Addenbrooke's Hospital, Hills Rd<br>Cambridge CB2 0QQ<br>UK Tel: +44 (0)1223 762 862<br>Fax: +44 (0)1223 330 598<br>E-mail: rh347@cam.ac.uk

This protocol has been written in accordance with ISO 14155:2020 standard

**Protocol Design**	Prof Roman Hovorka<br>University of Cambridge Metabolic Research Laboratories<br>Level 4, Wellcome Trust-MRC Institute of Metabolic Science<br>Box 289, Addenbrooke's Hospital, Hills Rd<br>Cambridge CB2 0QQ<br>UK Phone: +44 (0)1223 762 862<br>Fax: +44 (0)1223 330 598<br>E-mail: rh347@cam.ac.uk
**Principal Investigators**	Dr Charlotte Boughton<br>Institute of Metabolic Science<br>Addenbrooke's Hospital<br>Hills Road<br>Cambridge, CB2 0QQ<br>UK<br>Phone: +44 1223 769066<br>E-mail: cb2000@medschl.cam.ac.uk<br><br>Imperial College Healthcare NHS Trust<br>Department of Metabolism, Digestion and Reproduction, Faculty of Medicine<br>Hammersmith Campus<br>London, W12 0NN<br>UK<br><br>Manchester Royal Infirmary<br>Central Manchester University Hospitals NHS Foundation Trust<br>130, Hathersage Rd,<br>Manchester, M13 0JE<br>UK<br><br>King's College Hospital<br>King's College Hospital NHS Foundation Trust<br>Denmark Hill<br>London, SE5 9RS<br>UK<br><br>Norfolk and Norwich University Hospital<br>Norfolk & Norwich University Hospitals NHS Trust<br>Colney Lane, Norwich<br>Norfolk, NR4 7UY<br>UK

	Guy's & St Thomas' NHS Foundation Trust<br>200 Great Dover St,<br>London SE1 4YB<br>UK<br><br>Leicester Diabetes Centre<br>University Hospitals of Leicester NHS Trust<br>Gwendolen Rd,<br>Leicester, LE5 4PW<br>UK<br><br>University Hospitals of Derby and Burton NHS Foundation Trust<br>Uttoxeter Road<br>Derby, DE22 3NE<br>UK<br><br>Department of Diabetes, Endocrinology, Clinical Nutrition and Metabolism<br>Inselspital, Bern University Hospital<br>University of Bern<br>Bern<br>Switzerland<br><br>CHU de Toulouse<br>TSA 50032<br>31059 Toulouse Cedex 9<br>France<br><br>Prof.<br>Division of Endocrinology and Diabetology<br>Department of Medicine II<br>Medical Center – University of Freiburg<br>Hugstetter Str. 55<br>79106 Freiburg,<br>Germany

	Medical University of Graz,<br>Dept. of Internal Medicine<br>Division of Endocrinology and Diabetology<br>Auenbruggerplatz 15<br>A- 8036 Graz<br>Austria<br><br>Diabetes Centre, Institute of Clinical and Experimental Medicine,<br>Vídeňská 1958/9,<br>140 21 Prague,<br>Czech Republic.<br><br>Diabetes Technology Research Group<br>University of Melbourne Department of Medicine<br>St. Vincent's Hospital Melbourne<br>Level 4, Clinical Sciences Building<br>29 Princess Street<br>Fitzroy, Victoria<br>3065<br>Australia<br><br>Forschungsinstitut Diabetes-Akademie Bad Mergentheim (FIDAM GmbH)<br>Theodor Klotzbücher Str. 12<br>97980 Bad Mergentheim<br>Germany<br><br>Forschungsinstitut Diabetes-Akademie Bad Mergentheim (FIDAM GmbH)<br>Theodor Klotzbücher Str. 12<br>97980 Bad Mergentheim<br>Germany
**Other Investigators**	Institute of Metabolic Science<br>Box 289, Level 4<br>Addenbrooke's Hospital<br>Hills Road

	Cambridge, CB2 0QQ<br>UK<br><br>Institute of Metabolic Science<br>University of Cambridge<br>Box 289, Level 4<br>Addenbrooke's Hospital<br>Hills Road, Cambridge, CB2 0QQ<br>UK<br><br>Diabetes & Endocrinology Centre<br>North Manchester General Hospital<br>Delaunays Road<br>Manchester M8 5RB<br><br>Diabetes & Endocrinology Centre<br>North Manchester General Hospital<br>Delaunays Road<br>Manchester M8 5RB
**Study Co-ordinator**	Institute of Metabolic Science<br>University of Cambridge<br>Box 289, Level 4<br>Addenbrooke's Hospital<br>Hills Road, Cambridge, CB2 0QQ<br>UK
**Study Statistician**	Jaeb Center for Health Research<br>15310 Amberly Drive, Suite 350<br>Tampa, FL 33647<br>USA
**Human factors assessment**	Usher Institute<br>University of Edinburgh<br>Old Medical School<br>Teviot Place<br>Edinburgh, EH8 9AG

<table>
<tr><td></td><td colspan="2">UK

Usher Institute
University of Edinburgh
Old Medical School
Teviot Place
Edinburgh, EH8 9AG
UK</td></tr>
<tr><td rowspan="2">UK Study Sponsor</td><td colspan="2">Cambridge University Hospitals NHS Foundation Trust, jointly with University of Cambridge</td></tr>
<tr><td>Research Governance Office
University of Cambridge
School of Clinical Medicine
Box 111, Cambridge Biomedical Campus
Cambridge, CB2 0SP
UK</td><td>Cambridge University Hospitals
NHS Foundation Trust
Box 277, Addenbrooke's Hospital
Hills Road, Cambridge, CB2 0QQ,
UK</td></tr>
<tr><td>Switzerland Study Sponsor</td><td colspan="2">Inselspital, Bern University Hospital
University of Bern
Freiburgstrasse 18, 3010
Bern
Switzerland</td></tr>
<tr><td>France Study Sponsor</td><td colspan="2">University Hospital Toulouse, France
Hotel-Dieu, 2 rue Viguerie, TSA 800 35, 31059 Toulouse Cedex, France
Phone:
Email:</td></tr>
<tr><td>Germany Study Sponsor</td><td colspan="2">Forschungsinstitut Diabetes-Akademie Bad Mergentheim (FIDAM GmbH)
Theodor Klotzbücher Str. 12
97980 Bad Mergentheim
Germany</td></tr>
<tr><td>Austria Study Sponsor</td><td colspan="2">Medical University of Graz
Neue Stiftingtalstraße 6, 8010 Graz</td></tr>
</table>

**Czech Republic Study Sponsor**	Diabetes Centre, Institute of Clinical and Experimental Medicine,<br>Vídeňská 1958/9,<br>140 21 Prague,<br>Czech Republic.
**Australia Study Sponsor**	St. Vincent's Hospital Melbourne<br>Research Governance Unit<br>Level 1<br>93-103 Victoria Parade<br>Fitzroy, Victoria<br>3065<br>Australia

PROTOCOL SIGNATURE PAGE

The signature below documents the approval of the protocol entitled **“An open-label, multi-national, multi-centre, randomised, single-period parallel study to assess the efficacy, safety and utility of fully closed-loop insulin delivery compared to standard insulin therapy with CGM in adults with type 2 diabetes”.** Version 6.0 dated 14/10/2025 and provides the necessary assurances that this study will be conducted according to all stipulations of the protocol, the Medical Device Regulation (EU) 2017/745, ISO 14155:2020, the Declaration of Helsinki and local legal requirements.

Signature …………………………………….. Date………………………..

Prof Roman Hovorka, Coordinating Investigator

SITE SIGNATURE PAGE

I have read the attached protocol entitled **“An open-label, multi-national, multi-centre, randomised, single-period parallel study to assess the efficacy, safety and utility of fully closed-loop insulin delivery compared to standard insulin therapy with CGM in adults with type 2 diabetes”.** Version 6.0 dated 14/10/2025 and agree to abide by all provisions set forth therein.

I agree to comply with the Medical Device Regulation (EU) 2017/745, ISO 14155:2020, the Declaration of Helsinki and local legal requirements.

I agree to ensure that the confidential information contained in this document will not be used for any other purpose other than the evaluation or conduct of the clinical investigation without the prior written consent of the Sponsor.

Signature ……………………………………. Date……………………….

Name …………………………………….

Site, Country………………………….

Principal Investigator

Table of Contents

1. STUDY SYNOPSIS 14
LIST OF ABBREVIATIONS AND RELEVANT DEFINITIONS 21
2. SUMMARY 23
3. BACKGROUND 24
3.1. INTRODUCTION 24
3.2. GLYCAEMIC TARGETS IN PEOPLE WITH TYPE 2 DIABETES 24
3.3. CLOSED-LOOP INSULIN DELIVERY 25
3.4. CLOSED-LOOP RESEARCH IN CAMBRIDGE 26
3.4.1. DAY AND NIGHT CLOSED-LOOP STUDIES IN ADULTS WITH TYPE 1 DIABETES IN HOME SETTINGS 26
3.4.2. CLOSED-LOOP STUDIES IN ADULTS WITH TYPE 2 DIABETES IN THE INPATIENT SETTING 27
3.4.3. CLOSED-LOOP STUDIES IN ADULTS WITH TYPE 2 DIABETES IN THE HOME SETTING 28
3.5. RATIONALE FOR THE PRESENT STUDY 29
3.6. CAMAPS HX FULLY CLOSED-LOOP SYSTEM TO BE USED IN THE PRESENT STUDY 30
4. OBJECTIVES 31
4.1. EFFICACY 31
4.2. SAFETY 31
4.3. UTILITY 31
4.4. HUMAN FACTORS 31
5. STUDY DESIGN 31
6. STUDY PARTICIPANTS 33
6.1. STUDY POPULATION 33
6.1.1. INCLUSION CRITERIA FOR ALL COUNTRIES 33
6.1.2. EXCLUSION CRITERIA FOR ALL COUNTRIES 33
6.1.3. EXCLUSION CRITERIA SPECIFIC TO SWITZERLAND 34
6.1.4. EXCLUSION CRITERIA SPECIFIC TO FRANCE 34
6.1.5. EXCLUSION CRITERIA SPECIFIC TO GERMANY 34
6.2. RECRUITMENT AND INFORMED CONSENT 34
7. METHODS UNDER INVESTIGATION 36
7.1. NAME AND DESCRIPTION OF THE METHOD OF INVESTIGATION 36
7.2. INTENDED PURPOSE 36
7.3. METHOD OF ADMINISTRATION 36
7.4. REQUIRED TRAINING 36
7.5. PRECAUTIONS 37
7.6. ACCOUNTABILITY OF THE METHOD UNDER INVESTIGATION 37
8. STUDY SCHEDULE 38
8.1. OVERVIEW 38
8.2. VISIT 1 - RECRUITMENT VISIT AND SCREENING ASSESSMENT 41
8.3. VISIT 2 - REVIEW OF CGM DATA DURING RUN-IN PERIOD, COMPLIANCE ASSESSMENT AND RANDOMISATION 41
8.4. VISIT 3 - TRAINING AND INITIATION OF THE STUDY DEVICES 41
8.4.1. CLOSED-LOOP (INTERVENTION) 41
8.4.2. STANDARD INSULIN THERAPY WITH CGM (CONTROL) 42
8.5. TELEPHONE/EMAIL CONTACTS AFTER INITIATION OF TREATMENT ARM 43
8.6. VISIT 4 – 3 MONTH VISIT 43

8.7. Visit 5 – End of study visit ... 44
8.8. Participant withdrawal criteria ... 44
8.9. Study stopping criteria ... 45
8.10. Support telephone line ... 45
8.11. Participant reimbursement ... 45

9. ENDPOINTS ... 46

9.1. Efficacy endpoints ... 46
9.1.1. Primary efficacy endpoint ... 46
9.1.2. Other key endpoints ... 46
9.1.3. Secondary efficacy endpoints ... 46
9.1.4. Exploratory endpoints ... 46
9.2. Safety evaluation ... 47
9.3. Utility evaluation ... 47
9.4. Human factors evaluation ... 47

10. ASSESSMENT AND REPORTING OF ADVERSE EVENTS ... 48

10.1. Definitions ... 48
10.1.1. Reportable Adverse Events ... 48
10.1.2. Adverse Events ... 48
10.1.3. Adverse Device Effect ... 48
10.1.4. Serious Adverse Event ... 49
10.1.5. Serious Adverse Device Effect ... 49
10.1.6. Device Deficiencies ... 50
10.1.7. Adverse event intensity ... 50
10.1.8. Adverse event causality (MDCG 2020-10/1) ... 51
10.2. Recording and reporting of adverse events, serious adverse events and device deficiencies ... 51
10.2.1. Monitoring period of adverse events ... 51
10.2.2. Recording and reporting of adverse events ... 52
10.2.3. Severe hypoglycaemia ... 52
10.2.4. Reporting of serious adverse events and serious adverse device effects ... 53
10.2.4.1. UK ... 53
10.2.4.2. Switzerland ... 54
10.2.4.3. France ... 55
10.2.4.4. Germany ... 56
10.2.5. Recording and reporting of device deficiencies ... 58
10.2.6. Reporting of pregnancy ... 58
10.2.7. Healthcare arrangements and compensation for adverse events ... 58
10.3. Risks and anticipated adverse events and adverse device effects ... 60
10.3.1. Hypoglycaemia and hyperglycaemia ... 60
10.3.2. Blood sampling ... 60
10.3.3. Insulin injection therapy ... 60
10.3.4. Insulin pump therapy ... 60
10.3.5. Continuous glucose monitoring ... 61
10.3.6. Retinopathy ... 61
10.3.7. Questionnaires and interviews (participants) ... 61
10.3.8. Healthcare Professional Interviews (UK only) ... 62
10.3.9. Risk analysis and residual risk associated with the investigational device ... 62
10.4. Benefits ... 62
10.5. Burdens ... 63
10.6. Data Safety Monitoring Board (DSMB) ... 63

11. METHODS AND ASSESSMENTS ... 64

11.1. Procedures ... 64

11.1.1. Height, weight and blood pressure ... 64
11.1.2. Venepuncture ... 64
11.1.3. Urine Albumin Creatinine Ratio ... 64
11.1.4. Continuous subcutaneous glucose monitoring data ... 64
11.1.5. Insulin pump and closed-loop data ... 65
11.2. Assessment of Safety ... 65
11.3. Human factors assessments ... 65
11.3.1. Questionnaires ... 65
11.3.2. Participant Interviews ... 66
11.3.3 Healthcare Professional Interviews (UK only) ... 66

12. STUDY MATERIALS AND PRODUCTS ... 67

12.1. Insulin ... 67
12.2. Insulin injections ... 67
12.3. Insulin pump ... 67
12.4. Continuous subcutaneous glucose monitor ... 67
12.5. Smartphone ... 67
12.6. Computer-based algorithm ... 67

13. DATA ANALYSIS ... 69

13.1. Primary Endpoint Analysis ... 69
13.2. Key and secondary endpoint analysis ... 70
13.3. Safety Evaluation ... 71
13.4. Utility Evaluation ... 71
13.5. Questionnaires ... 71
13.6. Participant Interviews ... 72
13.8. Evaluative periods ... 72
13.9. Interim monitoring and analysis ... 72
13.10. Adherence and retention ... 73
13.11. Sample size and power calculations ... 73
13.12. Deviations from the statistical plan ... 73

14. CASE REPORT FORMS ... 73

15. DATA MANAGEMENT ... 74

15.1.1 Data security, access and back-up ... 75
15.1.2 Analysis and archiving ... 76
15.1.3 Electronic and central data validation ... 76

16. ETHICS ... 77

16.1. Research Ethics Committee and Institutional Review Board ... 77
16.2. Informed consent of study participants ... 77

17. AMENDMENTS TO THE PROTOCOL ... 78

18. DEVIATIONS FROM THE PROTOCOL ... 78

19. STUDY MANAGEMENT ... 79

19.1. Data and Safety Monitoring Board (DSMB) ... 79

20. RESPONSIBILITIES ... 79

20.1. Coordinating Investigator ... 79
20.2. Principal Investigator ... 79

21. REPORTS AND PUBLICATIONS ... 80

22. TIMETABLE ... 80

23. RETENTION OF STUDY DOCUMENTATION 80
24. INDEMNITY STATEMENTS 81
25. REFERENCES 83
26. DOCUMENT AMENDMENT HISTORY 87

1. Study Synopsis

**Title of clinical trial**	An open-label, multi-national, multi-centre, randomised, single-period parallel study to assess the efficacy, safety and utility of fully closed-loop insulin delivery compared to standard insulin therapy with CGM in adults with type 2 diabetes.
**Short title**	Closed-loop in adults with type 2 diabetes (COYOTE)
**Type of investigation (Switzerland only regulatory requirement)**	Clinical trial with a CE-marked medical device according to intended use (sub-category A1 according to Art 6 ClinO-MD).<br>Medical device: CamAPS HX, version 1.4<br>UDI: G008CAMAPSHX01BN<br>Manufacturer: CamDiab<br>SRN number: GB-MF-000001893 (Art 31 MDR)<br>Post market stage
**Sponsors name**	Cambridge University Hospitals NHS Foundation Trust and University of Cambridge, Cambridge, UK
**Medical condition or disease under investigation**	Type 2 diabetes
**Purpose of clinical trial**	To determine the efficacy, safety and utility of fully closed-loop insulin delivery over 26 weeks in the home setting in adults with type 2 diabetes.
**Study objectives**	The study objective is to compare fully closed-loop insulin delivery to standard insulin therapy with CGM over 26 weeks in adults with type 2 diabetes.<br>1. EFFICACY: The objective is to assess the ability of fully closed-loop insulin delivery to improve glucose control as measured by HbA1c (primary endpoint) and sensor glucose metrics.<br>2. SAFETY: The objective is to evaluate the safety of fully closed-loop insulin delivery in terms of episodes and severity of hypoglycaemia, and nature and severity of other adverse events.<br>3. UTILITY: The objective is to determine the acceptability and duration of use of the CGM and closed-loop system.

	4. HUMAN FACTORS: The objective is to assess cognitive, emotional, and behavioural characteristics of participants and their response to the closed-loop system using validated questionnaires and semi-structured interviews. Healthcare professionals working on the trial and supporting closed-loop users at UK sites will also be interviewed.
**Study design**	A multicentre, multinational, open-label, randomised single period, parallel design study contrasting fully closed-loop insulin delivery and standard insulin therapy with CGM over 26 weeks in adults with type 2 diabetes.
**Study endpoints**	The primary endpoint is the between group difference in centralised measurement of glycated haemoglobin (HbA1c) at 26 weeks.<br><br>Other key endpoints:<br>• Proportion of time spent in target glucose range (3.9 to 10.0mmol/l)*<br>• Mean sensor glucose*<br>• Proportion of time spent above target glucose (>10.0mmol/l)*<br>• Non-inferiority for time spent below target glucose (<3.9mmol/l)*<br><br>Secondary endpoints include:<br>• Standard deviation and coefficient of variation of glucose*<br>• Proportion of time with glucose <3.5mmol/l and <3.0mmol/l*<br>• Proportion of time with glucose >13.9mmol/l, >16.7mmol/l and >20.0mmol/l *<br>• Binary metrics for HbA1c (HbA1c <7.0% [53mmol/mol], HbA1c <7.5% [58.5mmol/mol])<br>• Total daily insulin dose<br>• Body weight, waist hip ratio and BMI<br>• Blood pressure<br>• Fasted lipid profile<br>• Renal function as measured by sodium, potassium, urea, serum creatinine, estimated Glomerular Filtration Rate (eGFR) and urinary albumin creatinine ratio (ACR)<br>• Liver function as measured by liver markers (ALT, AST, ALP, yGT, bilirubin and albumin) and FIB4 index

	*Glucose related endpoints based on CGM data over 26 weeks
**Safety evaluation**	Assessment of frequency and severity of hypoglycaemic episodes and nature and severity of other adverse events.
**Utility evaluation**	Assessment of the acceptability and duration of use of the CGM and closed-loop system.
**Human factors evaluation**	Assessment of cognitive, emotional, and behavioural characteristics of participants and their response to the closed-loop system using validated surveys and semi-structured interviews. Healthcare professionals working on the trial and supporting closed-loop users at UK sites will also be interviewed.
**Participating clinical centres**	UK<br>1. Addenbrooke's Hospital, Cambridge University Hospitals NHS Foundation Trust.<br>2. Imperial College Healthcare NHS Trust, London<br>3. Manchester University NHS Foundation Trust<br>4. King's College Hospital, King's College Hospital NHS Foundation Trust, London<br>5. Guy's and St Thomas' NHS Foundation Trust<br>6. Norfolk and Norwich University Hospital, Norfolk and Norwich University Hospitals NHS Foundation Trust<br>7. University Hospitals of Leicester NHS Trust<br>8. University Hospitals of Derby and Burton NHS Foundation Trust<br>Switzerland<br>9. Inselspital, Bern University Hospital, Bern<br>France<br>10. Centre Hospitalier Universitaire (CHU) de Toulouse<br>Germany<br>11. Medical Center – University of Freiburg<br>12. Forschungsinstitut Diabetes-Akademie Bad Mergentheim (FIDAM GmbH)<br>Austria<br>13. Medical University of Graz, Graz<br>Czech Republic<br>14. Diabetes Centre, Institute for Clinical and Experimental Medicine, Prague<br>Australia

	15. St Vincent's Hospital, Melbourne, Australia
**Sample size**	224 participants (112 per group) will be randomised. Recruitment will target a minimum quota of 25% of participants using basal insulin and a minimum quota of 60% of participants using multiple daily insulin injections.
**Summary of eligibility criteria**	Key inclusion criteria:<br>1. Aged 18 years and older<br>2. Type 2 diabetes diagnosed for at least 12 months<br>3. Established on an SGLT2 inhibitor and/or GLP-1 receptor agonist for at least 3 months, or have been offered these therapies previously.<br>4. Treatment with insulin therapy for at least 6 months<br>5. HbA1c ≤ 15% (140 mmol/mol) analysis from local laboratory or equivalent<br>6. Willing to wear study devices and follow study instructions<br>7. Capacity to consent to participate in the study<br><br>Key exclusion criteria:<br>1. Type 1 diabetes<br>2. Current use of insulin pump<br>3. Current use of any closed-loop system<br>4. Any physical/psychological disease or medication(s) likely to interfere with the conduct of the study and interpretation of the study results, as judged by study clinician<br>5. Known or suspected allergy against insulin<br>6. Pregnancy, planned pregnancy, or breast feeding<br>7. Severe visual impairment<br>8. Severe hearing impairment<br>9. Medically documented allergy towards the adhesive (glue) of plasters<br>10. Serious skin diseases located at places of the body, which potentially are possible to be used for localisation of the glucose sensor<br>11. Illicit drugs abuse<br>12. Prescription drugs abuse<br>13. Alcohol abuse
**Maximum duration of study for a participant**	30 weeks
**Recruitment**	Participants will be recruited through outpatient diabetes clinics, primary care centres, social media advertising or other established methods at participating centres in:

	• UK<br>• Switzerland<br>• France<br>• Germany<br>• Austria<br>• Czech Republic<br>• Australia
**Consent**	Participants will be asked to provide written informed consent.
**Screening and baseline assessment**	Eligible participants will undergo baseline evaluation involving taking a medical history including demographics, height/weight, waist hip ratio and blood pressure measurement and blood samples for HbA1c, fasted lipid profile, renal and liver function. A urine albumin-creatinine ratio (ACR) will be performed, along with a urine pregnancy test in females of child-bearing age. Human factors questionnaires will be completed and a masked glucose sensor will be applied.
**Run-in Period**	During a 2-3 week run-in period, participants will use their usual insulin therapy and wear a masked CGM system. At the end of the run-in period, for compliance, at least 10 days of CGM data needs to be recorded.<br>CGM data during the run-in period will be used to assess baseline glucose control before the start of the intervention phase.
**Randomisation**	Eligible participants will be randomised in a 1:1 ratio using central randomisation software to fully closed-loop or standard insulin therapy with CGM for 26 weeks. Randomisation will be stratified by site and baseline HbA1c.
**1. Closed loop arm**	Following randomisation, participants in the closed-loop group will receive training on the study CGM, study insulin pump and closed-loop App during a 1-2 hour outpatient session. Competency on the use of the closed-loop system will be evaluated. Further training may be delivered as required. Participants will be advised to use the closed-loop system for the next 26 weeks. A subset of participants will participate in an interview sub-study.

**2. Standard therapy with CGM (control arm)**	Following randomisation, participants in the control group will use their usual insulin therapy and the study CGM. Training on the use of the CGM will be provided. Participants will use standard insulin therapy and CGM for the next 26 weeks.
**3-month study visit**	Weight, waist hip ratio and blood pressure will be measured and a blood sample will be taken for measurement of HbA1c, fasted lipid profile, renal and liver function. Data from the closed-loop system and CGM system will be reviewed. Human factors questionnaires will be completed.
**Other study contacts**	Participants will be contacted by the study team (email/phone) monthly to record any adverse events, device deficiencies, and changes to medications.
**End of study assessments**	Weight, waist hip ratio and blood pressure will be measured and a blood sample will be taken for measurement of HbA1c, fasted lipid profile, renal and liver function. Urinary ACR will be measured. Human factors questionnaires will be completed and a subset of participants will participate in an interview study. Study devices will be returned and participants will resume their usual insulin therapy and standard glucose monitoring.
**Procedures for safety monitoring during trial**	Standard operating procedures for monitoring and reporting of all adverse events and adverse device events will be in place, including serious adverse events (SAE), serious adverse device effects (SADE) and specific adverse events (AE) such as severe hypoglycaemia.<br>A data safety and monitoring board (DSMB) will be informed of all serious adverse events and any unanticipated serious adverse device effects that occur during the study and will review compiled adverse event data at periodic intervals.
**Criteria for withdrawal of patients on safety grounds**	A participant may terminate participation in the study at any time without necessarily giving a reason and without any personal disadvantage. An investigator can stop the participation of a participant after consideration of the benefit/risk ratio. Possible reasons are:<br>1. Participant is unable to demonstrate safe use of study devices as judged by the investigator

	2. Serious adverse events<br>3. Significant protocol violation or non-compliance<br>4. Decision by the investigator, or the Sponsor, that termination is in the participant's best medical interest<br>5. Pregnancy, planned pregnancy, or breast feeding<br>6. Allergic reaction to insulin or severe allergic reaction to adhesive surface of infusion set or glucose sensor<br>7. Technical grounds (e.g. participant relocates)<br><br>Efforts will be made to retain participants in follow up for the final primary outcome assessment even if the intervention is discontinued, unless the investigator believes that it will be harmful for the participant to continue in the trial.

List of abbreviations and relevant definitions

ACCORD	Action to Control Cardiovascular Risk in Diabetes
ACR	Albumin Creatinine Ratio
ADE	Adverse Device Effect
ADVANCE	Action in Diabetes and Vascular Disease: Preterax and Diamicron MR Controlled Evaluation
AE	Adverse Event
AP	Artificial Pancreas
AR	Adverse Reaction
ASADE	Anticipated Serious Adverse Device Effect
AUC	Area Under the Curve
BMI	Body Mass Index
CE	Conformité Européenne (CE-mark)
CGM	Continuous Glucose Monitoring
CI	Coordinating Investigator *or* Confidence Interval
CL	Closed Loop
CRF	Case Report Form
CSII	Continuous Subcutaneous Insulin Infusion
DD	Device Deficiency
DKA	Diabetic Ketoacidosis
DSMB	Data Safety and Monitoring Board
eGFR	Estimated Glomerular Filtration Rate
GCP	Good Clinical Practice
HbA1c	Glycated haemoglobin A1c
HFS	Hypoglycaemia Fear Survey
MDI	Multiple Daily Injection therapy
MHRA	Medicine and Healthcare products Regulatory Agency
MPC	Model-Predictive-Control
NHS	National Health Service
PI	Principal Investigator
R&D	Research and Development

RCT	Randomised Controlled Trial
REC	Research Ethics Committee
SADE	Serious Adverse Device Effect
SAE	Serious Adverse Event
SAP	Sensor Augmented Pump Therapy
SD	Standard Deviation
T1D	Type 1 Diabetes Mellitus
T2D	Type 2 Diabetes Mellitus
UKPDS	UK Prospective Diabetes Study
USADE	Unanticipated Serious Adverse Device Effect
VADT	Veterans Affairs Diabetes
WHO	World Health Organisation
WHR	Waist Hip Ratio

2. Summary

The main objective of this study is to determine the efficacy, safety and utility of fully closed-loop glucose control in the home setting in adults with type 2 diabetes (T2D). This study builds on previous and on-going studies of closed-loop systems that have been performed in Cambridge in adults with type 2 diabetes in the inpatient and in the home setting and in children and adults with type 1 diabetes.

This is an open-label, multi-national, multi-centre, randomised, single-period parallel study, involving a run-in period followed by a 26-week intervention period during which glucose levels will be controlled either by a fully closed-loop system or by participants usual insulin therapy with continuous glucose monitoring. A total of up to 224 adults with type 2 diabetes using insulin will be recruited through outpatient diabetes clinics, primary care centres, social media advertising and other established methods at participating centres. Participants will receive appropriate training in the safe use of the study devices.

The primary outcome is the between group difference in HbA1c at 26 weeks. Other key outcomes include the time spent with glucose levels within, above and below the target glucose range (3.9-10.0mmol/L) and mean sensor glucose as recorded by CGM over the 26 weeks. Insulin requirements, body weight, renal and liver function will also be compared. Safety evaluation comprises severe hypoglycaemic episodes, and other adverse and serious adverse events. Human factors outcomes include CGM & closed-loop usage, questionnaires and semi-structured interviews with participants, and interviews with healthcare professionals supporting closed-loop users (UK sites only).

3. Background

3.1. Introduction

Type 2 diabetes (T2D) is a condition characterised by chronic hyperglycaemia due to impairments in insulin secretion, insulin action, or both (1). The burden of T2D is widespread with an estimated 415 million people affected worldwide, which is expected to double within the next 20 years (2). Although more commonly seen in the older population, incidence of T2D is also increasing in younger age groups due to rising obesity levels from physical inactivity and calorie dense diet (3).

It is estimated that annual global health expenditure on diabetes is 760 million USD and 50% of this is spent on treating complications of the disease (4). The fear of long-term complications and concerns about managing the condition is a significant burden for patients and healthcare professionals. It is well established that lowering of HbA1c to <7% (53mmol/mol) reduces microvascular complications of type 1 and 2 diabetes (5). With the rising prevalence of T2D across all age groups, it is important that therapies are optimised to achieve the best possible blood glucose control to prevent long term complications of the disease.

Lifestyle interventions have shown an element of reversibility in some people with T2D and is important in reducing cardiovascular risk (6), however the majority of people with T2D ultimately require a combination of lifestyle interventions and pharmacological therapy as the disease progresses. An increasing number of oral diabetes medications and non-insulin injectable therapies have become available to target different mechanisms driving hyperglycaemia in T2D. Side effect profiles, contraindications, or the progressive nature of the disease itself often results in the need to intensify treatment to insulin therapy (7). In the UK Prospective Study (UKPDS), over 50% of the patients with newly diagnosed T2D required additional insulin therapy alongside oral diabetes medications within six years (8). Treatment with insulin in T2D has been shown to be effective at improving glycaemic control, however can lead to problematic hypoglycaemia (9). This limitation to insulin therapy may provide an explanation as to why only approximately 50% of people with T2D reach the recommended target HbA1c (2).

The use of fully closed-loop technology to enable safe intensification of insulin in this population is a promising and realistic therapeutic approach.

3.2. Glycaemic targets in people with type 2 diabetes

Current National Institute for Health and Care Excellence (NICE) guidelines support an HbA1c target of 48mmol/mol (6.5%) for adults with T2D managed by lifestyle and diet ± a single medication not

associated with hypoglycaemia. A target of 53mmol/mol (7%) is recommended for patients on a medication associated with hypoglycaemia. Intensification of therapy is recommended at HbA1c 58mmol/mol (7.5%) or above and it is at this stage that insulin is considered (10).

A ten year follow up of the UKPDS study showed continued reduction in microvascular risk with intensive glucose management in patients with T2D (11), therefore glucose lowering has remained central to management guidelines. The traditional aim to achieve 'near normal' HbA1c in patients with T2D, however, was challenged in the ACCORD (Action to Control Cardiovascular Risk in Diabetes) and VADT (Veterans Affairs Diabetes) trials, where there was no significant reduction seen in cardiovascular events and increased mortality with intensive therapy (target HbA1c <6%) compared to standard therapy. Among the postulated causes for this included a higher incidence of severe hypoglycaemic events along with weight gain and the use of multiple drugs to achieve this level of control in the ACCORD trial. In contrast, patients in the ADVANCE (Action in Diabetes and Vascular Disease: Preterax and Diamicron MR Controlled Evaluation) trial achieved a similar median HbA1c to ACCORD within the intensive treatment arm but with no increase in mortality and a key difference noted between the two studies was a higher rate of severe hypoglycaemia in the ACCORD trial (3% vs 16%) (5), suggesting that avoidance of hypoglycaemia is crucial when intensifying therapy.

A meta-analysis in 2015 showed that, on average, a patient with T2D on insulin experiences 23 episodes of mild-moderate hypoglycaemia (defined as no third-party assistance required) and 1 episode of severe hypoglycaemia (requiring third-party assistance), per year. This was higher than in patients who were on oral agents alone (12). A closed-loop system represents a major step towards safer and more effective insulin administration in this population.

3.3. Closed-loop insulin delivery

The emergence of new technologies including continuous glucose monitors (CGM) (13), insulin pumps and sensor augmented pump therapy (SAP) (14-16) provides new opportunities to improve outcomes for people with diabetes. The most promising approach is closed-loop insulin therapy (17) which combines real-time CGM with insulin pump therapy to achieve glucose-responsive subcutaneous insulin delivery. The vital component of such a system, also known as an artificial pancreas (AP), is a control algorithm. The role of the control algorithm is to compute the amount of insulin to be delivered by the pump using the real-time sensor glucose levels.

The closed-loop approach has been successfully evaluated in children and adults with type 1 diabetes in controlled laboratory studies (18-20) and in home settings (21-26). The results

demonstrated improved glucose control and reduced risk of hypoglycaemia events. Psychosocial assessments supported acceptability and positive impact of this approach and hybrid closed-loop systems are now transforming the management of type 1 diabetes. A fully closed-loop approach to the management of type 2 diabetes has been evaluated in the inpatient setting (27-29), and in the home setting (30, 31), with results suggesting that this technology is a tangible option to improve glucose control in this population.

3.4. Closed-loop research in Cambridge

The University of Cambridge and collaborators have a considerable track record investigating closed-loop glucose control in young children, older children, adolescents, adults, and pregnant women with type 1 diabetes (21, 32-35) and in adults with type 2 diabetes (27-31).

Studies that have been performed employed model predictive control (MPC) – this algorithm estimates user-specific parameters from CGM measurements taken every 1 to 15 minutes and makes predictions of glucose excursions, which are then used to direct insulin infusion (36). To date, trials in people with type 1 diabetes have used a hybrid closed-loop system, where the standard bolus calculator is used to deliver prandial insulin. Trials in people with type 2 diabetes have used a fully closed-loop algorithm, where insulin delivery is fully automated with no mealtime bolusing.

3.4.1. Day and night closed-loop studies in adults with type 1 diabetes in home settings

Following closed-loop studies supervised in the clinical research facility, and overnight in the home setting, in 2014 we completed a first study testing a day and night home system over a seven day period in 17 adults. This randomised clinical trial adopted a multicentre, multi-national, crossover design. During the home phase, the percentage time when glucose was in target range (3.9 to 10.0mmol/l) was significantly higher during closed-loop compared to sensor augmented pump therapy (75 [61, 79] vs. 62 [53, 70]%, median [IQR], p=0.005). Mean glucose (8.1 vs. 8.8 mmol/l, p=0.027) and time spent above target (p=0.013) were lower during closed-loop while time spent below target was comparable (p=0.339). Increased time in target was observed during both day-time (p=0.017) and night-time (p=0.013).

We completed a multicentre, multinational, crossover, randomised, controlled study under free living home conditions for 12 weeks comparing 24/7 closed-loop insulin delivery with sensor augmented pump therapy (control intervention) in 33 adults with type 1 diabetes (22). The proportion of time

that the glucose level was in the target range (3.9 to 10.0mmol/l) was 11.0 percentage points (95% confidence interval [CI], 8.1 to 13.8) greater with the use of the closed-loop system day and night than with control therapy (P<0.001). The mean glucose level was lower during the closed-loop phase than during the control phase (difference, −0.6 mmol/l; 95% CI, −0.9 to −0.3; P<0.001), as were the area under the curve for the period when the glucose level was less than 3.5 mmol/l (39% lower; 95% CI, 24 to 51; P<0.001) and the mean glycated haemoglobin level (difference, −0.3%; 95% CI, −0.5 to −0.1; P = 0.002).

In a 12 week open-label, multicentre, multinational (UK and USA), single period, parallel study (APCam11) (34), we analysed data from children and adults with sub-optimally controlled (HbA1c 7.5-10%) type 1 diabetes with 24/7 CL (n=46) or SAP (n=40; control) over 12 weeks of unrestricted living. CL increased time glucose was in target range by 13±8 percentage points compared with a 2±6 percentage point increase in control group (primary endpoint; p<0.001). In the closed-loop group, HbA1c was reduced from screening value of 8.3±0.6% to 8.0±0.6% post run-in and 7.4±0.6% post intervention. In the control group these values were 8.2±0.5%, 7.8±0.6% and 7.7±0.5%; reductions in A1c levels were significantly greater in closed loop group compared to control group (mean difference in change 0.4%; 95% CI, 0.2% to 0.6%; p<0.001). Mean sensor glucose was lower in closed loop group (p<0.001) as was the time spent with sensor glucose levels below 3.9 mmol/l (p=0.008) and above 10.0 mmol/l (p<0.001).

In an open-label, multicentre, multinational (UK and Austria), crossover study (37), we compared data from 31 adults with well-controlled type 1 diabetes (HbA1c <7.5%) with 24/7 CL or CSII (control) over 4 weeks of unrestricted living. The proportion of time when sensor glucose was in target range was 10.5 percentage points higher (95% CI 7·6-13·4; p<0·0001) during closed-loop delivery compared with usual pump therapy (65.6% [SD 8.1] when participants used usual pump therapy vs 76.2% [6.4] when they used closed-loop). Compared with usual pump therapy, closed-loop delivery also reduced the proportion of time spent in hypoglycaemia: the proportion of time with glucose concentration below 3.5 mmol/L was reduced by 65% (53-74, p<0·0001) and below 2.8 mmol/L by 76% (59-86, p<0·0001). No episodes of serious hypoglycaemia or other serious adverse events occurred.

3.4.2. Closed-loop studies in adults with type 2 diabetes in the inpatient setting

The Cambridge fully closed-loop system has been shown to be safe and feasible in insulin naïve patients with type 2 diabetes (T2D) in a clinical research facility setting (38). We have previously assessed fully closed-loop insulin delivery in non-critical care patients with T2D hospitalised in the general wards (28, 39). In a multi-national study, 136 adults with T2D who required insulin therapy

were randomised to receive either closed-loop insulin delivery or conventional subcutaneous insulin therapy for up to 15 days or until hospital discharge (28). The mean percentage of time that the sensor glucose measurement was in the target range was 65.8% in the closed-loop group and 41.5% in the control group, a difference of 24.3 percentage points (P<0.001); values above the target range were found in 23.6% and 49.5% of the patients, respectively, a difference of 25.9 percentage points (P<0.001). The mean glucose level was 8.5 mmol/l in the closed-loop group and 10.4 mmol/l in the control group (P<0.001). There was no significant between-group difference in the duration of hypoglycaemia or in the amount of insulin that was delivered. Among inpatients with T2D receiving noncritical care, the use of a fully automated, closed-loop insulin-delivery system resulted in significantly better glycaemic control than conventional subcutaneous insulin therapy, without a higher risk of hypoglycaemia.

We have also assessed fully automated closed-loop insulin delivery in non-critical care patients requiring insulin therapy who were receiving enteral or parenteral nutritional support. Forty-three adults were randomised to receive either closed-loop insulin delivery or conventional subcutaneous insulin therapy for up to 15 days or until hospital discharge (29). Results showed that closed-loop increased time spent within target glucose range (3.9-10.0mmol/l) from 36.4% to 68.4%, a difference of 32 percentage points (p<0.001). This showed that fully-closed loop insulin delivery is an effective treatment option to improve glycaemic control in inpatients receiving nutritional support.

3.4.3. Closed-loop studies in adults with type 2 diabetes in the home setting

In an open-label, multinational (UK and Switzerland), randomised, crossover study we enrolled 27 adults with T2D using insulin therapy and end-stage renal failure requiring maintenance dialysis (31). Participants received fully closed-loop insulin therapy with faster-acting insulin aspart or their standard insulin therapy for a period of 20 days in the home setting. The order of the two interventions was random. The proportion of time sensor glucose was in target range of 5.9-10.0mmol/L was 53% during closed-loop and 38% during control therapy, a difference of 15 percentage points which equates to an additional 3.5 hours each day spent in target glucose range with closed-loop. Mean sensor glucose was lower in the closed loop group (p=0.002) as was the time spent with sensor glucose levels below 3.9 mmol/L (p=0.040). There was no difference in the amount of insulin delivered (P=0.38). One episode of severe hypoglycaemia occurred during the closed-loop period but closed-loop was not in operation at the time.

More recently, we have assessed fully closed-loop insulin delivery in the wider population with T2D requiring insulin. In a single-centre, randomized crossover study, 26 adults with T2D underwent two 8-week periods comparing fully closed-loop insulin therapy with standard insulin therapy and a

masked glucose sensor in random order (30). The proportion of time in target glucose range (3.9-10.0mmol/l) was 66% with closed-loop therapy v 32% with control therapy, a difference of 35 percentage points which equates to an additional 8 hours each day spent in target glucose range with closed-loop. Mean glucose was lower during the closed-loop therapy period than during the control therapy period (9.2 mmol/l v 12.6 mmol/L, P < 0.001). HbA1c was lower following closed-loop therapy compared to following control therapy (57mmol/mol v 72 mmol/mol, P < 0.001). There was no increase in time in hypoglycaemia (<3.9mmol/l). Fully closed-loop insulin delivery, with no user input required at mealtimes, improved glucose control without increasing hypoglycaemia compared with standard insulin therapy and may represent a safe and efficacious method to improve outcomes in adults with T2D.

3.5. Rationale for the present study

The prevalence of T2D is increasing, and few people with T2D achieve recommended glycaemic targets (2). This leads to increasing burden of diabetes management and complications for patients and also negatively impacts on resource requirements and the budget for health and care services supporting people with T2D.

We have recently shown that a fully closed-loop system can dramatically improve glucose control in adults with T2D, without increasing the risk of hypoglycaemia (30). This novel therapeutic approach may also improve health-related quality-of-life for people with T2D, and reduce the risk of long-term complications. We anticipate that this approach may also reduce the need for healthcare provider input for treatment optimisation.

The present trial will assess whether fully closed-loop insulin delivery for 26 weeks improves glucose control compared to standard insulin therapy with a glucose sensor in a larger and more diverse cohort of adults with T2D. This research will provide important evidence to determine whether fully closed-loop technology is a cost-effective approach which can provide sustained health benefits for people with T2D, and could influence recommendations for widespread clinical adoption and reimbursement.

3.6. CamAPS HX fully closed-loop system to be used in the present study

In the present study, we will use the fully closed-loop system comprising:

- YpsoPump insulin pump (Ypsomed, Switzerland)
- FreeStyle Libre 3 real-time CGM sensor (Abbott Diabetes Care, CA, USA) or an alternative compatible CE-marked CGM system
- Smartphone hosting CamAPS HX App (CamAPS HX, CamDiab, UK) with the Cambridge model predictive control algorithm
- Glooko (Glooko, Inc, CA, USA) Cloud upload system to review CGM/insulin data.

An overview of this closed loop system is given in Figure 1.

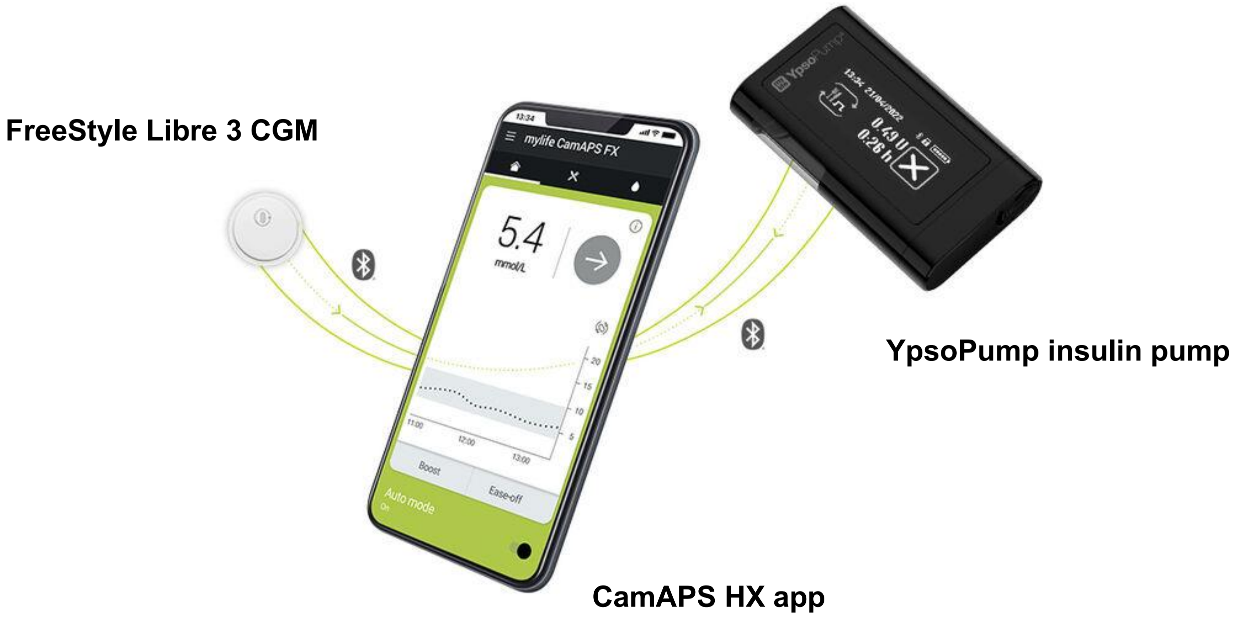

Figure 1: The fully closed-loop system comprises the CamAPS HX app (CamDiab) running on a smartphone, YpsoPump insulin pump (Ypsomed), Freestyle Libre 3 real-time CGM sensor (Abbott Diabetes Care).

4. Objectives

4.1. Efficacy

To assess the efficacy of fully closed-loop therapy in improving glucose control as measured by glycated haemoglobin and sensor glucose metrics, as compared to standard insulin therapy with CGM.

4.2. Safety

To evaluate the safety of fully closed-loop therapy in terms of episodes and severity of hypoglycaemia and nature and severity of other adverse events.

4.3. Utility

To determine the acceptability and duration of use of the CGM and closed-loop system.

4.4. Human Factors

To assess cognitive, emotional, and behavioural characteristics of participants and their response to the closed-loop system using questionnaires and semi-structured interviews. Healthcare professionals working on the trial and supporting closed-loop users at UK sites will also be interviewed.

5. Study Design

A multi-national, multi-centre randomised, open-label, single period, parallel design study of fully closed-loop insulin delivery compared to standard insulin therapy with a sensor over 6 months in adults with T2D.

The study flow chart is outlined in Figure 2.

Figure 2: Study flow chart

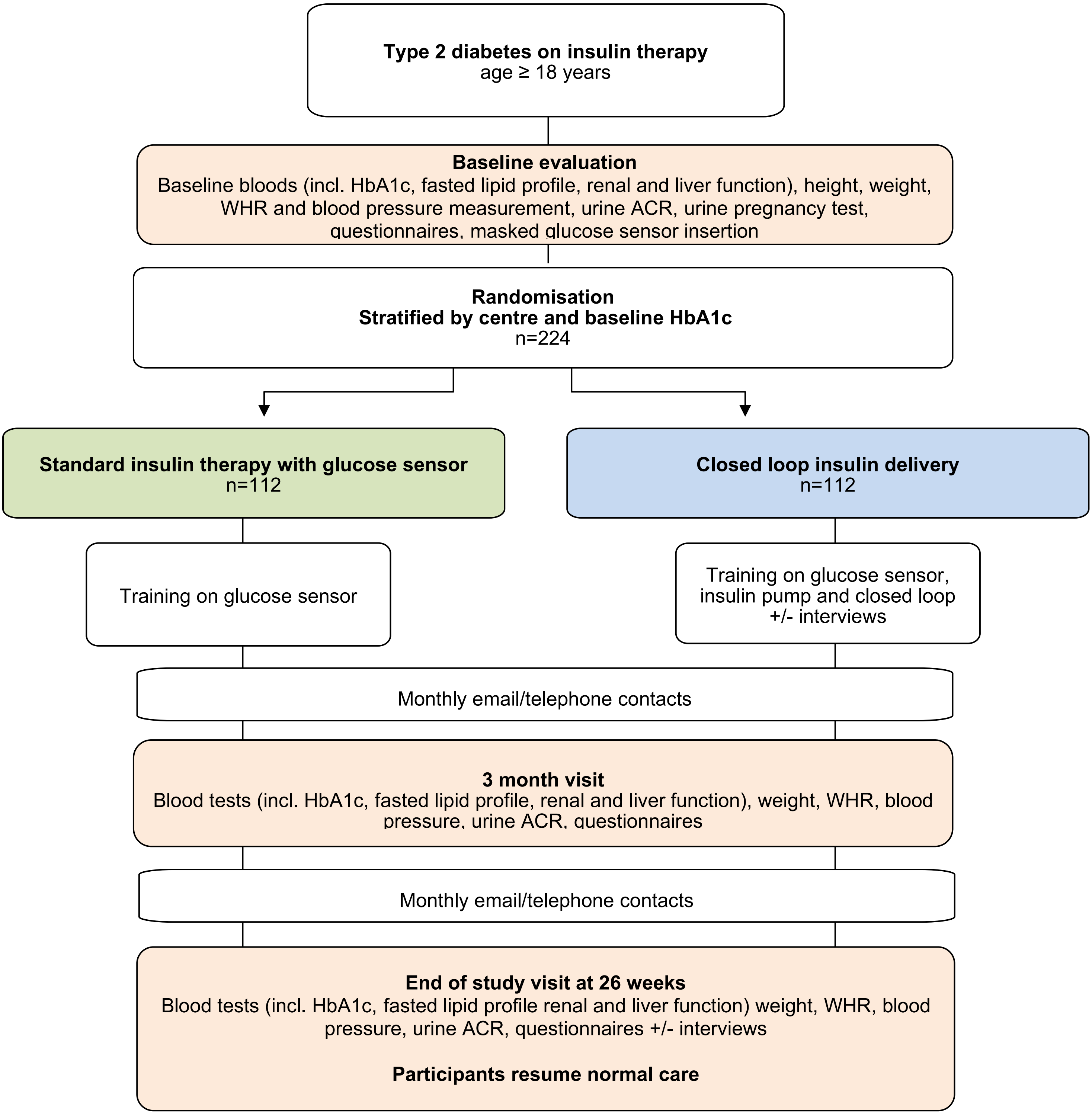

6. Study participants

6.1. Study Population

Adults aged 18 years and over with type 2 diabetes requiring insulin therapy will be recruited.

6.1.1. Inclusion criteria for all countries

1. Aged 18 years and older
2. Type 2 diabetes diagnosed for at least 12 months
3. Established on an SGLT2 inhibitor and/or GLP-1 receptor agonist for at least 3 months, or have been offered these therapies previously.
4. Treatment with insulin therapy for at least 6 months
5. HbA1c ≤ 15% (140 mmol/mol) analysis from local laboratory or equivalent
6. Willing to wear study devices and follow study instructions
7. Capacity to consent to participate in the study

6.1.2. Exclusion criteria for all countries

1. Type 1 diabetes
2. Current use of insulin pump
3. Current use of any closed-loop system
4. Any physical/psychological disease or medication(s) likely to interfere with the conduct of the study and interpretation of the study results, as judged by study clinician
5. Known or suspected allergy against insulin
6. Pregnancy, planned pregnancy, or breast feeding
7. Severe visual impairment
8. Severe hearing impairment
9. Medically documented allergy towards the adhesive (glue) of plasters
10. Serious skin diseases located at places of the body, which potentially are possible to be used for localisation of the glucose sensor
11. Illicit drugs abuse
12. Prescription drugs abuse
13. Alcohol abuse

Potential participants with diabetes related retinal disease will not be excluded from the trial. An individualised risk approach will be used, and we will consider delaying recruiting people undergoing active treatment of proliferative retinopathy / maculopathy until treatment is complete.

6.1.3. Exclusion criteria specific to Switzerland

1. Lack of safe contraception for female participants of childbearing potential for the entire study duration
2. Incapacity to give informed consent
3. Not literate in German

6.1.4. Exclusion criteria specific to France

1. Lack of safe contraception for female participants of childbearing potential for the entire study duration
2. Incapacity to give informed consent
3. Not literate in French
4. Patient participating in another research protocol including an exclusion period still in progress
5. Patient under the protection of justice or guardianship

6.1.5. Exclusion criteria specific to Germany

1. Lack of safe contraception for female participants of childbearing potential for the entire study duration
2. Incapacity to give informed consent
3. Not literate in German

6.1.6. Exclusion criteria specific to Australia

1. Incapacity to give informed consent
2. Patient participating in another research protocol including an exclusion period still in progress

6.2. Recruitment and informed consent

Recruitment will target up to 224 participants to allow for drop-outs and to increase the number of participants who will be exposed to the closed-loop system for an enhanced safety and feasibility

assessment. Recruitment will target a minimum quota of 25% of participants using basal insulin and a minimum quota of 60% of participants using multiple daily insulin injections.

Participants will be recruited from outpatient diabetes clinics, primary care centres, social media advertising or other established methods at each of the following sites.

1. Addenbrooke's Hospital, Cambridge University Hospitals NHS Foundation Trust, Cambridge, UK
2. Imperial College Healthcare NHS Trust, London, UK
3. Central Manchester University Hospitals NHS Foundation Trust, Manchester, UK
4. King’s College Hospital, King’s College NHS Foundation Trust, London, UK
5. Guy's and St Thomas' NHS Foundation Trust, London, UK
6. Norfolk and Norwich University Hospital, Norfolk and Norwich University Hospitals NHS Foundation Trust, UK
7. University Hospitals of Leicester NHS Trust, UK
8. University Hospitals of Derby and Burton NHS Foundation Trust, UK
9. Inselspital, Bern University Hospital, Bern, Switzerland
10. Centre Hospitalier Universitaire (CHU) de Toulouse, France
11. Medical Center – University of Freiburg, Germany
12. Medical University of Graz, Graz, Austria
13. Diabetes Centre, Institute for Clinical and Experimental Medicine, Prague, Czech Republic
14. St Vincent’s Hospital Melbourne, Victoria, Australia
15. Forschungsinstitut Diabetes-Akademie Bad Mergentheim (FIDAM GmbH), Bad Mergentheim, Germany

Local GP surgeries in the region of the study site will be included as Participant Identification Centres (PICs) for the UK study sites.

Each centre will aim to recruit between 15-50 participants. Methods of patient recruitment will follow established practices at each centre. Potential participants will be given the study information leaflet and invited to join the study by the research team at least one week before the recruitment visit.

Written informed consent will be obtained from all participants before any study related activities.

7. Methods under Investigation

7.1. Name and description of the method of investigation

The investigational treatment is the CamAPS HX closed-loop app as described in section 3.6 manufactured by CamDiab, Cambridge, UK. The CamAPS HX app is being used in accordance with the instructions for use.

7.1.1 Switzerland requirement only

The making available on the market, the putting into service or the use of the product to be investigated is not prohibited in Switzerland.
The subjects concerned are not subjected to any additional invasive or stressful procedures compared with those applied under normal condition of use of the product (subcategory A1).

7.2. Intended purpose

The intended purpose of the investigational treatment is automated fully closed-loop insulin delivery. The investigated medical device is used to manage glucose levels in adults with type 2 diabetes, using a fully closed-loop approach.

7.3. Method of administration

The closed-loop system consists of components directly attached to the patient, which are the CGM sensor and the insulin pump. The component not directly attached to the patient is the handheld smartphone that contains the closed-loop algorithm and communicates wirelessly with the insulin pump (Figure 1).

7.4. Required training

Prior to commencement of the study, the research team nurses/clinicians at each of the investigation centres will be trained to use the closed-loop system and its components. Prior to the use of study devices, participants randomised to closed-loop will be trained to use the study CGM device, the study pump and the closed-loop app. Participants randomised to standard insulin therapy will be trained to use the study CGM device. Training will be provided during a 1-2 hour session as an outpatient. Competency assessments of the participants' capability to safely use study devices will be undertaken before the participant is allowed to start using the devices at home.

7.5. Precautions

During treatment with insulin there is a risk of hypoglycaemia and hyperglycaemia. In-hospital testing and hazard analysis have documented reduced risk of hypoglycaemia and hyperglycaemia during closed-loop compared to conventional treatment.

7.6. Accountability of the method under investigation

The local investigators will provide training for the study participants and will make every effort, through regular contact, to ascertain that the closed-loop system is used for the study purposes only. Devices will be identified using serial numbers and the location of investigational devices and their dates of use by participants will be documented throughout the study.

8. Study schedule

8.1. Overview

The study will be coordinated from the Institute of Metabolic Science, Addenbrookes Hospital, Cambridge, UK and performed at the following sites:

1. Addenbrooke's Hospital, Cambridge University Hospital NHS Foundation Trust, UK
2. Imperial College Healthcare NHS Trust, London, UK
3. Manchester University Hospitals NHS Foundation Trust, UK
4. King’s College Hospital, London, UK
5. Guy's and St Thomas' NHS Foundation Trust, London, UK
6. Norfolk and Norwich University Hospitals, UK
7. University Hospitals of Leicester NHS Trust, UK
8. University Hospitals of Derby and Burton NHS Foundation Trust, UK
9. Inselspital, Bern University Hospital, Switzerland
10. Centre Hospitalier Universitaire (CHU) de Toulouse
11. Medical Center – University of Freiburg, Germany
12. Medical University of Graz, Graz, Austria
13. Diabetes Centre, Institute for Clinical and Experimental Medicine, Prague, Czech Republic
14. St Vincent’s Hospital Melbourne, Australia
15. Forschungsinstitut Diabetes-Akademie Bad Mergentheim (FIDAM GmbH), Bad Mergentheim, Germany

After recruitment, consent, and a 2-3 week run-in period, participants will be randomised to 26 weeks use of closed-loop insulin delivery or 26 weeks during which they will apply their standard insulin therapy with real-time continuous glucose monitoring.

The study includes up to 5 visits and 6 telephone/email contacts. Visits 2 and 3 may be combined. All participants will continue to be seen by their clinical team at frequencies as appropriate in line with usual clinical practice. All study visits will be scheduled in addition to routine clinical visits and will be performed by the research team. Where possible, for convenience, dates for study visits will be arranged along with routine clinic appointments. Maximum time in the study is 30 weeks. At the end of the study (completion of the final study visit), all participants will return the study devices and resume their pre-study treatment.

Table 1 outlines study activities when the participant is randomised to closed-loop (intervention group).

Table 2 outlines study activities when the participant is randomised to standard insulin therapy + CGM (control group).

Table 1. Schedule of study visits / contacts when the participant is randomised to closed-loop (intervention group).

	**Visit/ contact**	**Description**	**Start relative to previous / next visit / activity**	**Duration**
**Baseline & Run-in**	**Visit 1**	Recruitment visit: Consent, baseline bloods (inc HbA1c, fasted lipid profile, renal and liver function) height, weight, WHR and BP, urine ACR, urine pregnancy test, questionnaires, masked CGM insertion		1-2 hours
	**Visit 2**	Review of baseline bloods and CGM data. Randomisation	2-3 weeks after Visit 1 (±1 week);	30 minutes
**Post-randomisation Training**	**Visit 3**	Study pump, CGM and closed-loop training and initiation, competency assessment	May coincide with Visit 2, within 2-4 weeks of Visit 1	1-2 hours
**CL Intervention (6 months)**	Contact 1	Review use of study devices; study update	within 48 hours after Visit 3	30 minutes
	Contact 2	Review use of study devices; study update	1 week after Visit 3 (±3 days)	30 minutes
	Contact 3	Review use of study devices; study update	1 month after Visit 3 (±2 weeks)	30 minutes
	Contact 4	Review use of study devices; study update	2 months after Visit 3 (±2 weeks)	30 minutes
	**Visit 4**	3-month visit, HbA1c, fasted lipid profile, renal and liver function, weight, WHR, BP, urine ACR, questionnaires	3 months after Visit 3 (±2 weeks)	1 hour
	Contact 5	Review use of study devices; study update	4 months after Visit 3 (±2 weeks)	30 minutes
	Contact 6	Review use of study devices; study update	5 months after Visit 3 (±2 weeks)	30 minutes
	**Visit 5**	End of closed-loop treatment arm; bloods (HbA1c, fasted lipid profile renal and liver function), weight, WHR, BP, urine ACR, questionnaires +/- interviews; resume usual care	6 months after Visit 3 (±2 weeks)	1-2 hours

Table 2. Schedule of study visits / contacts when the participant is randomised to standard insulin therapy + CGM (control group).

	Visit/ contact	Description	Start relative to previous / next Visit / Activity	Duration
**Baseline and run-in**	**Visit 1**	Recruitment visit/ screening assessment: Consent, baseline bloods (inc HbA1c, fasted lipid profile, renal and liver function) height, weight, WHR and BP, urine ACR, urine pregnancy test, questionnaires, masked CGM insertion		1-2 hours
	**Visit 2**	Review of baseline bloods and CGM data. Randomisation	2-3 weeks after Visit 1 (±1 week);	30 minutes
**Post-randomisation training**	**Visit 3**	CGM training and initiation, competency assessment	May coincide with Visit 2, within 2-4 weeks of Visit 1	1-2 hours
**Standard insulin + CGM (Control) (6 months)**	Contact 1	Review use of study devices; study update	within 48 hours after Visit 3	30 minutes
	Contact 2	Review use of study devices; study update	1 week after Visit 3 (±3 days)	30 minutes
	Contact 3	Review use of study devices; study update	1 month after Visit 3 (±2 weeks)	30 minutes
	Contact 4	Review use of study devices; study update	2 months after Visit 3 (±2 weeks)	30 minutes
	**Visit 4**	3-month visit, HbA1c, fasted lipid profile, renal and liver function, weight, WHR, BP, urine ACR, questionnaires	3 months after Visit 3 (±2 weeks)	1 hour
	Contact 5	Review use of study devices; study update	4 months after Visit 3 (±2 weeks)	30 minutes
	Contact 6	Review use of study devices; study update	5 months after Visit 3 (±2 weeks)	30 minutes
	**Visit 5**	End of standard insulin + CGM treatment arm; bloods (HbA1c, fasted lipid profile, renal and liver function), weight, WHR, BP, urine ACR, questionnaires; resume usual care	6 months after Visit 3 (±2 weeks)	1-2 hours

8.2. Visit 1 - Recruitment visit and screening assessment

Potential participants will be given a verbal explanation of the study and will have the opportunity to ask any questions. If they agree to proceed with the study, and providing they meet the inclusion/exclusion criteria, participants will give written informed consent to a member of the research team. Those who would prefer to have more time to consider their decision to participate can return and give consent at a later date.

At the screening visit, participant's height, weight, waist hip ratio, blood pressure, demographics, medical and diabetes history, concomitant medications and insulin therapy will be recorded. Participants will have blood tests including HbA1c, fasted lipid profile, renal function and liver function. Urine albumin creatinine ratio (ACR) will be measured. A urine pregnancy test will be performed in all females aged between 18 and 60 years. A masked continuous glucose monitor (CGM) will be applied to assess baseline glycaemic control and participants will be asked to complete questionnaires to assess quality of life and diabetes management.

Participants will then proceed to the run-in period (2-3 weeks) where they will use their usual insulin therapy and wear the masked CGM. For those already using CGM as part of their usual care, they can continue with their usual CGM and also wear the masked study CGM. There will be a minimum of two weeks run-in period for all participants (end of Visit 1 to Visit 2).

8.3. Visit 2 - Review of CGM data during run-in period, compliance assessment and randomisation

At the end of the run-in period (2-3 weeks after Visit 1), participants CGM data will be reviewed. A minimum of 10 days of data recorded will be needed to proceed with randomisation. This visit can be done remotely if preferred.

Eligible participants will be randomised in a 1:1 ratio using a centrally administered web-based randomisation programme to the use of fully closed loop or to standard insulin therapy with CGM for 26 weeks. The randomisation will be stratified by clinical centre and baseline HbA1c.

8.4. Visit 3 - Training and initiation of the study devices

8.4.1. Closed-loop (intervention)

This session will be conducted by a pump educator and/or member of the study team. This visit can be done at the clinical research facility or outpatient department. Study training, where preferred,

can be supported by online training modules and webinars which have been developed for the closed-loop system.

Participants will receive training to cover key aspects of insulin pump use, CGM and closed-loop insulin delivery. Particular attention will be paid to:

- Insulin cartridge and infusion set changes and correct priming procedure
- Insertion and initiation of sensor session
- The impact of diet and lifestyle on glucose sensor readings and trends
- Hypo- and hyperglycaemia management
- Setting and adjustment of alarms/alerts
- Connection and disconnection of the closed-loop system

Written easy to use guidelines for the operation of the insulin pump, CGM and closed-loop system will be provided. Competency in the use of study pump, CGM and closed-loop system will be assessed by the study team. Only participants who demonstrate competency in use of the system will be allowed to continue to the home study phase.

Closed-loop glucose control will start once the training session is completed. Participants will be advised to continue closed-loop over the next 26 weeks. Participants will be asked to contact the study team if they encounter any problems with the closed-loop system.

All non-insulin diabetes therapies will be continued throughout the study period. Throughout the trial, participants and/or the clinical team are free to adjust diabetes therapy as per usual clinical practice.

A subset of participants (n=30, recruited from across UK sites) randomised to the closed-loop group will be invited to take part in a qualitative interview to explore their experiences of using the closed-loop system. These interviews will take place near the end of their participation in the trial.

8.4.2. Standard insulin therapy with CGM (control)

This session will be conducted by a pump educator and/or member of the study team. This visit can be done at the clinical research facility or outpatient department. Study training, where preferred, will be supported by online training modules and webinars developed for the CGM system.

Participants will receive training to cover key aspects of the study CGM device. Particular attention will be paid to:

- Insertion and initiation of sensor session

- Interpreting real-time CGM data and trend arrows
- The impact of diet and lifestyle on glucose sensor readings and trends
- Setting and adjustment of alarms/alerts
- Managing hypo- and hyperglycaemia

Written easy to use guidelines for the operation of the CGM device will be provided. Competency on the use of CGM will be assessed by the study team. Only participants who demonstrate competency on use of the CGM will continue to the home study phase.

Standard insulin therapy with CGM will start once the training session is completed. Participants will be advised to use their usual insulin therapy with CGM over the next 26 weeks. Participants will be asked to contact the study team if they encounter any problems with the CGM system.

All non-insulin diabetes therapies will also be continued throughout the study period. Throughout the trial, participants and/or the clinical team are free to adjust diabetes therapy as per usual clinical practice.

8.5. Telephone/email contacts after initiation of treatment arm

All participants will be contacted by email / telephone within 48 hours and at one week after initiation of the respective study arm. The purpose of this contact would be to troubleshoot any issues with the devices, and to record any adverse events, device deficiencies, and changes in insulin doses, other medical conditions and/or medication.

Thereafter, participants will be followed up through study contacts (telephone / email) at monthly intervals to record any adverse events, device deficiencies, changes in insulin doses, other medical conditions and/or medication.

8.6. Visit 4 – 3 month visit

Participants will attend the research centre for Visit 4 approximately three months after Visit 3. Participants will have a blood test for HbA1c, fasted lipid profile, renal function and liver function. Participant’s weight, waist hip ratio and BP will be recorded and urine ACR measured. Participants will be asked to complete questionnaires to assess quality of life and diabetes management.

8.7. Visit 5 – End of study visit

The participant will attend the research centre approximately six months after Visit 3. This will be the end of study visit. Participants will have a blood test for HbA1c, fasted lipid profile, renal function and liver function. Participant's weight, waist hip ratio and BP will be recorded and urine ACR measured. Participants will be asked to complete questionnaires to assess quality of life and diabetes management.

At the end of study visit, study devices will be collected and participants will resume their usual diabetes care.

A subset of participants will be invited to take part in a qualitative interview to assess quality-of-life impacts of using the closed-loop system.

8.8. Participant withdrawal criteria

The following pre-randomisation withdrawal criterion will apply:

1. Participant is unable to demonstrate safe application of insulin therapy as judged by the investigator.

The following pre- and post-randomisation withdrawal criteria will apply:

2. Participant is unable to demonstrate safe use of insulin injections or study insulin pump, CGM and/or closed-loop during post randomisation training period as judged by the investigator.
3. Participants may terminate participation in the study at any time without necessarily giving a reason and without any personal disadvantage
4. Significant protocol violation or non-compliance
5. Recurrent severe hypoglycaemia events related to the use of the closed-loop system
6. Recurrent severe persistent hyperglycaemia events/DKA unrelated to infusion site failure and related to use of the closed-loop system
7. Decision by the investigator or the Sponsor that termination is in the participant's best medical interest
8. Allergic reaction to insulin, or severe reaction to adhesive surface of infusion set or glucose sensor

Participants who are withdrawn for reasons stated in (2) to (8) will be invited to provide a blood sample and complete questionnaires at the end of the planned study intervention.

8.9. Study stopping criteria

The study may be stopped if three consecutive participants withdraw from the study for safety reasons, or on the advice of the independent Data Safety Monitoring Board (DSMB).

8.10. Support telephone line

There will be a telephone helpline for the local research team to access advice in case of any technical issues.

8.11. Participant reimbursement

The study will provide the CGM device, insulin pump, smartphone (where required) and related consumables. A study payment will be made to reflect local practice. The amount paid will be specified in the participant information sheet and REC application form. Reasonable travel expenses will be reimbursed. After completing the study, participants will not keep the study devices. They will revert to their usual insulin therapy and glucose monitoring.

8.12. Healthcare Professional Interviews (UK only)

Interviews will be conducted with healthcare professionals (n=20, 2-3 per site) involved in trial recruitment and delivery, recruited from UK-based sites. Interviews will be scheduled near the end of the trial and/or when the healthcare professional stops working on it (e.g., due to changing job), to maximise the wealth of experience of supporting closed-loop system use upon which interviewees can draw. Interviews will be informed by topic guides which will be revised in light of emerging findings, in line with an inductive approach.

9. Endpoints

9.1. Efficacy endpoints

9.1.1. Primary efficacy endpoint

The primary endpoint is the between group difference in centralised measurement of glycated haemoglobin (HbA1c) at 26 weeks.

9.1.2. Other key endpoints

- Proportion of time spent in the target glucose range (3.9 to 10.0mmol/l)*
- Mean sensor glucose*
- Proportion of time spent above target glucose (>10.0mmol/l) *
- Non-inferiority for time spent below target glucose (<3.9mmol/L) *

9.1.3. Secondary efficacy endpoints

- Standard deviation and coefficient of variation of glucose*
- Proportion of time with glucose <3.5mmol/l and <3.0mmol/l*
- Proportion of time with glucose >13.9 mmol/l, >16.7mmol/l and >20.0mmol/l *
- Binary metrics for HbA1c (HbA1c <7.0%[53mmol/mol], HbA1c <7.5%[58.5mmol/mol])
- Total daily insulin dose
- Body weight, waist hip ratio and Body Mass Index
- Fasted lipid profile
- Renal function as measured by sodium, potassium, urea, serum creatinine, estimated Glomerular Filtration Rate and urinary albumin creatinine ratio
- Liver function as measured by liver markers (ALT, AST, ALP, yGT, bilirubin and albumin) and FIB4 index

*Glucose related endpoints based on CGM data over 26 weeks

9.1.4. Exploratory endpoints

- Day (06:00 to 23:59) and night (00:00 to 05:59) glucose control
- Monthly trends in glucose control and insulin delivery

9.2. Safety evaluation

Safety evaluation will comprise the number of episodes of severe hypoglycaemia, as well as nature and severity of any other adverse events including SAEs, SADEs and AEs over 26 weeks. All participants including those who withdraw will be included in the safety evaluation.

9.3. Utility evaluation

Utility evaluation is the acceptability and duration of use of the CGM and closed-loop system over the 26 week intervention periods.

9.4. Human factors evaluation

Expectations, attitudes and responses to the closed-loop system will be assessed using validated questionnaires (perceptions of quality of life, burden of diabetes management, and fear of hypoglycaemia) as well as qualitative interviews with selected participants and healthcare professionals supporting closed-loop users (UK sites only).

10. Assessment and Reporting of Adverse Events

10.1. Definitions

10.1.1. Reportable Adverse Events

A reportable Adverse Event is any untoward medical occurrence that meets criteria for a serious adverse event or any unanticipated medical occurrence in a study participant that is study or device-related. Device deficiencies that could have led to a serious adverse device effect will also be reported (ISO 14155: 8.2.5 and 9.8).

10.1.2. Adverse Events

An adverse event (AE) is any untoward medical occurrence, unintended disease or injury, or untoward clinical signs (including abnormal laboratory findings) in a participant, users or other persons, who has received an investigational device, whether or not related to the investigational medical device (ISO 14155: 3.2). This definition includes events related to the device under investigation or the comparator or to the study procedures. The following anticipated adverse events will not be recorded:

- Non clinically significant skin reactions as judged by investigator
- Pre-existing medical conditions
- New illnesses or conditions not requiring concomitant medication or medical intervention/procedures
- Non severe hypoglycaemia
- Hyperglycaemia without significant ketonaemia (<3mmol/L)

10.1.3. Adverse Device Effect

An Adverse Device Effect (ADE) is an adverse event related to the use of an investigational medical device (ISO 14155: 3.1). This includes adverse events resulting from insufficient or inadequate instructions for use, deployment, implantation, installation, or operation, or any malfunction of the investigational medical device. This definition also includes any event resulting from use error or from intentional misuse of the device under investigation.

10.1.4. Serious Adverse Event

A serious adverse event (SAE, ISO 14155, 3.45) is an adverse event that:

- led to a death
- led to a serious deterioration in the health of the participant, users, or other persons that either resulted in:
    - a life threatening illness or injury
    - a permanent impairment of a body structure or function including chronic diseases
    - in-patient hospitalisation or prolonged hospitalisation
    - medical or surgical intervention to prevent life-threatening illness or injury or permanent impairment to a body structure or a body function
- led to foetal distress, foetal death or a congenital abnormality or birth defect including physical or mental impairment

A planned hospitalisation for pre-existing condition, or a procedure required by the study protocol, without a serious deterioration in health, is not considered to be a serious adverse event.

More than one of the above criteria can be applicable to one event. Life-threatening in the definition of a serious adverse event or serious adverse reaction refers to an event in which the participant was at risk of death at the time of the event; it does not refer to an event which hypothetically might have caused death if it were more severe. Medical judgement should be exercised in deciding whether an adverse event or reaction is serious in other situations.

Important adverse events or reactions that are not immediately life-threatening or do not result in death or hospitalisation but may jeopardise the participant or may require intervention to prevent one of the other outcomes listed in the definition above, should also be considered serious.

10.1.5. Serious Adverse Device Effect

A Serious Adverse Device Effect (SADE; ISO 14155, 3.44) is an adverse device effect that has resulted in any of the consequences characteristic of a serious adverse event.

- An Unanticipated Serious Adverse Device Effect (USADE) is a serious adverse device effect which by its nature, incidence, severity or outcome has not been identified in the current version of the protocol or the risk analysis report (ISO 14155: 3.51).
- An Anticipated Serious Adverse Device Effect (ASADE) is a serious adverse device effect which by its nature, incidence, severity or outcome has been identified in the protocol or the risk analysis report.

10.1.6. Device Deficiencies

A device deficiency is an inadequacy of a medical device with respect to its identity, quality, durability, reliability, usability, safety or performance, of an investigational device, including malfunction, user errors and inadequate information supplied by the manufacturer (Art. 2 Abs 59 MDR, ISO 14155: 3.19). Malfunction is the failure of an investigational device to perform in accordance with its intended purpose when used in accordance with the instructions for use (ISO14155: 3.33). The definition includes deficiencies related to the investigational medical device or the comparator medical device.

A device deficiency may lead to an Adverse Device Effect or Serious Adverse Device Effect (Art. 80 Abs 1 letter c MDR; ISO 14155). This includes any device deficiency that might have led to a serious adverse event if appropriate action had not been taken, intervention had not occurred, or circumstances had been less fortunate.

The following anticipated device deficiencies and device-related issues will not be recorded:

- Infusion set occlusion/leakage not leading to ketonaemia (<3mmol/L)
- Sensor failure due to significant over/under-reading (difference>3mmol/L) or detachment
- Premature interruption of sensor-life
- Battery lifespan deficiency due to inadequate charging or extensive wireless communication
- Closed-loop system error messages not needing system replacement
- Intermittent device communication failure not leading to system replacement

10.1.7. Adverse event intensity

Intensity	Definition
Mild	Patient is aware of signs and symptoms but they are easily tolerated
Moderate	Signs / symptoms cause sufficient discomfort to interfere with usual activities
Severe	Patient is incapable to work or perform usual activities

NB. The term “severe” is often used to describe the intensity (severity) of a specific event. This is not the same as ‘serious’, which is based on patient/event outcome or action criteria (see definition 10.1.4). For example, itching for several days may be rated as severe, but may not be clinically serious.

10.1.8. Adverse event causality (MDCG 2020-10/1)

Intensity	Definition
Not related	The relationship to the device or procedures can be excluded.
Possible	The relationship with the use of the investigational device is weak but cannot be ruled out completely. Alternative causes are also possible.
Probable	The relationship with the use of the investigational device seems relevant and/or the event cannot reasonably be explained by another cause.
Causal Relationship	The serious event is associated with the investigational device or with procedures beyond reasonable doubt.

10.2. Recording and reporting of adverse events, serious adverse events and device deficiencies

10.2.1. Monitoring period of adverse events

The period during which adverse events will be reported is defined as the period from the beginning of the study (obtaining informed consent) until 2 weeks after the end of the study participation (completion of the final study visit). Adverse events that continue after the participant’s discontinuation or completion of the study will be followed until their medical outcome is determined or until no further change in the condition is expected. The follow up of AEs may therefore extend after the end of the clinical investigation; however no new AEs will be reported after the trial reporting period.

10.2.2. Recording and reporting of adverse events

Throughout the course of the study, all efforts will be made to remain alert to possible adverse events or untoward findings. The first concern will be the safety of the participant, and appropriate medical intervention will be taken. The investigator will elicit reports of adverse events from the participant at each visit and complete adverse event forms. All AEs, including those the participant reports spontaneously, those the investigators observe, and those the participant reports in response to questions will be recorded on paper or electronic AE forms at each site within seven days of discovering the event. Furthermore, device deficiencies (DD) will also be collected, fully investigated and documented in the source document and appropriate CRF during the entire investigation period, i.e. from patient's informed consent until the final study visit.

- Documentation of AEs (including SAEs) by the PI includes diagnosis or symptoms, start and stop dates of event, event treatment, event resolution, assessment of seriousness and causal relationship to medical device and/or investigation procedure (ISO14155).
- Documentation of DDs by the PI includes description of event, start date, investigational device information, action taken with regard to the investigational device, and whether the DD led to an AE. The Sponsor shall review all DDs and determine and document in writing whether they could have led to a SAE (DD with SADE potential) (ISO14155).

The study investigator will assess the relationship of any adverse event to be device-related or unrelated by determining if there is a reasonable possibility that the adverse event may have been caused by the study device or study procedures. The individual investigator at each site will be responsible for managing all adverse events according to local protocols, and decide if reporting is required.

10.2.3. Severe hypoglycaemia

Severe hypoglycaemia will be defined as an event requiring assistance of another person due to altered consciousness to actively administer carbohydrate, glucagon, or other resuscitative actions. This means that the participant is impaired cognitively to the point that he/she is unable to treat him or herself, is unable to verbalize his or her needs, is incoherent, disoriented, and/or combative, or experiences seizure or coma. If plasma glucose measurements are not available during such an event, neurological recovery attributable to the restoration of plasma glucose to normal is considered sufficient evidence that the event was induced by a low plasma glucose concentration.

Severe hypoglycaemia will be regarded as a foreseeable adverse event and an adverse event form will be completed. Severe hypoglycaemia is not necessarily a serious adverse event and hence may

not require immediate reporting to the Sponsor. Non-severe hypoglycaemia will not be reported or considered an adverse event.

10.2.4. Reporting of serious adverse events and serious adverse device effects

When reporting adverse events, all pertinent data protection legislation must be adhered to.
The serious adverse event report should contain the following information*:

1. Study identifier (EudraCT number if applicable)
2. Participant's unique study number
3. Date of birth
4. Event description
5. Start date of event
6. Laboratory tests used and medical interventions used to treat the SAE
7. Planned actions relating to the event, including whether the study device was discontinued
8. Statement on the patient's current state of health
9. Criterion for seriousness (i.e. death, life threatening, hospitalisation, disability/incapacity or other)
10. Evaluation of causality (including grade of relatedness) with the following (more than one may apply):
    a. the investigational treatment/medical device
    b. the clinical study/a study specific procedure
    c. other: e. g. concomitant treatment, underlying disease
11. Date of procedure
12. Reporter's name, date and signature

*In the case of incomplete information at the time of initial reporting, all appropriate information should be provided as soon as this becomes available.

The relationship of the SAE to the investigational treatment / medical device should be assessed by the investigator at site, as should the anticipated or unanticipated nature of any SAEs and SADEs.

10.2.4.1. UK

All SAEs whether or not deemed investigational method/device related and whether anticipated or unanticipated must be reported to the Sponsor by email or fax within 24 hours (one working day) of the Investigator learning of its occurrence.
SAEs should be reported to:
Stephen Kelleher
Cambridge University Hospitals

NHS Foundation Trust
Box 277, Addenbrooke's Hospital
Hills Road, Cambridge, CB2 0QQ, UK
Phone: +44 (0) 1223 217418
Fax: +44 (0) 1223 348494
E-mail: cuh.research@nhs.net

A written report must follow within five working days and is to include a full description of the event and sequelae, in the format detailed on the Serious Adverse Event reporting form. If applicable, the Sponsor will notify the competent authority of all Serious Adverse Events in line with pertinent legal requirements.

The Investigator will notify the Research Ethics Committee (REC) in UK of all Serious Adverse Events in line with pertinent legal requirements. The Investigator will inform the Sponsor about all reports sent to the reporting organisation including follow-up information and answers by the reporting organisation. The local principal investigator is responsible for informing other site principal investigators and the CI of all SAEs.

The regulatory authority (MHRA) will be notified of all SAEs as soon as possible within ten days of the event occurring during the study. The REC will be notified of all unexpected and related SAEs within 15 days of the occurrence of the event.

10.2.4.2. Switzerland

All SAEs, device deficiencies and health hazards that require measures are reported to the Principal Investigator by the study team within 24 hours after becoming aware of the event. Device deficiencies are assessed regarding their potential to lead to an SAE. SADE and device deficiencies with SADE potential will be reported to the Coordinating Investigator by the study team within 3 days. SAEs will evaluated by the Sponsor and Principal Investigator with regard to causality and seriousness.

They should be reported to:

Dr Lia Bally
Department of Diabetes, Endocrinology, Clinical Nutrition and Metabolism
Inselspital, Bern University Hospital
University of Bern, Bern
Switzerland

Tel: 0041 31 632 36 77
Email: lia.bally@insel.ch

The Sponsor and Principal Investigator reports to the Ethics Committee promptly any serious adverse event which has a causal relation with the medical device, comparator or procedure/test method or where a causal relation appears to be possible (Art. 33 ClinO-MD).
In order to ensure prompt notification, the Sponsor and Principal Investigator can initially submit an incomplete notification.
If safety and health hazards that require measures must be taken immediately during the conduct of the investigation, the Sponsor and Principal Investigator notifies the Ethics Committee within 2 days of these measures and the circumstances which made them necessary (Art. 34 ClinO-MD).
SADE and device deficiencies with SADE potential will be reported to the Sponsor-Investigator by the study team within 7 days.
All SADEs, DD with SADE potential and safety measures will also be notified via the local materiovigilance system to Swissmedic.
Pregnancies will be reported within a maximum of 24 hours to the Sponsor-Investigator and result in withdrawal of the study subject without any follow-up procedures due to lack of safety-concerns in the event of pregnancy occurrence.
An Annual Safety Report (ASR) is submitted by the Sponsor and Principal Investigator to the Ethics Committee, yearly (Art. 35 ClinO-MD). The ASR contains a list of all SADEs and DDs and a report on their degree of seriousness, causal relationship with the MD and procedure and on subjects' safety.
An End of study notification will be sent 15 days after the end of study to the Ethics Committee and a final report will be sent to the Ethics Committee 1 year after study completion. Other reporting will be done according to provisions of MD vigilance as per Art. 87-90 MDR (Art. 33 para 4.b ClinO-MD) and Art. 67 MedDO.

Any interruption will be communicated within 15 days to the Ethics Committee after interruption (if interruption is safety-related: within 24hrs).

10.2.4.3. France

All serious adverse events will be managed and reported in compliance with all applicable regulations and included in the final clinical study report.

10.2.4.4. Germany

The reporting of serious adverse events (SAEs) and product defects (DDs) must be carried out in accordance with the European Regulation 2017/745 (MDR) in conjunction with the Medical Device Law Implementation Act (MPDG) and must be applied for or reported to the higher federal authority.

Any reportable event as defined in Article 80 MDR or Section 64(1) MPDG that indicates an immediate risk, death, serious injury, or serious illness and that requires immediate corrective action for other patients/proband, users, or other persons, or that involves new knowledge should be reported promptly, but no later than 2 calendar days after the Sponsor becomes aware of a new reportable event or new information related to a previously reported event.

All other reportable events described in Article 80 MDR or Section 64 Section 1 MPDG, or a new finding/update thereto should be reported immediately, but no later than 7 calendar days after the date the sponsor becomes aware of the new reportable event or new information related to a previously reported event.

All SAEs, device deficiencies and health hazards that require measures must be reported by the study investigators to the Sponsor within 24 hours after the SAE becomes known using the "Serious Adverse Event" form.

Device deficiencies are assessed regarding their potential to lead to an SAE. SAEs will be evaluated by the Sponsor and Principal Investigator with regard to causality and seriousness.

Pregnancies will be reported within a maximum of 24 hours to the Sponsor-Investigator and result in withdrawal of the study subject without any follow-up procedures due to lack of safety-concerns in the event of pregnancy occurrence.

SAEs should be reported to:
University Hospital Freiburg
Klinik für Innere Medizin II – Studienbüro
Hugstetter Str. 55
79106 Freiburg,
Germany
Phone: +49-761-270-39591 Fax +49-761-270-96 33730
E-mail: med.2-gi-study@uniklinik-freiburg.de

10.2.4.5. Austria

The Sponsor shall be informed by the investigator immediately, but not later than 3 calendar days after investigation site study personnel’s awareness of the event.

Report by Sponsor to National Competent Authority (NCA)

For all reportable events which indicate an imminent risk of death, serious injury, or serious illness and that requires prompt remedial action for other patients/subjects, users or other persons or a new finding to it: Immediately, but not later than 2 calendar days after awareness by sponsor of a new reportable event or of new information in relation with an already reported event. This includes events that are of significant and unexpected nature such that they become alarming as a potential public health hazard. It also includes the possibility of multiple deaths occurring at short intervals. These concerns may be identified by either the NCA or the manufacturer.

Any other reportable events or a new finding/update to it: Immediately, but not later than 7 calendar days following the date of awareness by the sponsor of the new reportable event or of new information in relation with an already reported event.

Report by the Sponsor to the ethics committee

Reportable events that occurred within the domestic territory (Austria) have to be reported to the concerned ethics committee by the sponsor.

10.2.4.6. Czech Republic

All SAEs and SADEs will be reported to the Sponsor by the study investigators within 24 hours (1 working day) of learning of their occurrence. All SAEs and SADEs will also be reported to the regulatory authorities (State Drug Agency of the Czech Republic) in an expedited manner (within 24 hours) of their reporting to the Sponsor. All adverse events will be managed and reported in compliance with all applicable regulations, and included in the final clinical study report.

SAEs/SADEs should be reported to:

Prof. Martin Haluzik
Diabetes Center
Institute for Clinical and Experimental Medicine
Videnska 1958/9
140 21 Prague 4, Czech Republic
Phone: +420 236 054 108
E-mail: halm@ikem.cz

10.2.4.7. Australia

The study clinician will immediately report to the Sponsor any SAEs, whether or not considered study intervention related, including those listed in this protocol or IB, and must include an assessment of whether there is a reasonable possibility that the study intervention caused the event. All SAEs will be reported immediately to the HREC at St Vincent's Hospital Melbourne and within 2 working days of Sponsor awareness to the Australian medicine and therapeutic regulatory agency The Therapeutics Goods Administration (TGA) (where appropriate).

The TGA require that all serious and unexpected adverse device events are reported to the Devices Clinical Section, Office of Blood, Devices and Tissues of the TGA in an expedited fashion (i.e. within 15 calendar days of first knowledge), or for fatal or life-threatening events, an initial or full report within 7 calendar days and a follow-up report if necessary within the 15 calendar day timeframe.

10.2.5. Recording and reporting of device deficiencies

All device deficiencies will be documented throughout the study. The investigator at each site will be responsible for managing all device deficiencies and determine and document in writing whether they could have led to a serious adverse device effect.

All device deficiencies that might have led to a serious adverse device effect(s) if: suitable action had not been taken; intervention had not been made, or if circumstances had been less fortunate, must be reported to the Sponsor as for SAEs/SADEs.

10.2.6. Reporting of pregnancy

Study participants will not routinely be tested for pregnancy as part of the trial follow up process. Any pregnancy which does occur during the course of the study should be reported to the Sponsor as indicated in section 10.2.4 immediately. In the UK it is at the investigator's discretion to decide whether the individual should be instructed to stop study treatment.

10.2.6.1 Austria

The absence of pregnancy by urine pregnancy test must be determined before and once a month during the clinical trial.

10.2.7. Healthcare arrangements and compensation for adverse events

Healthcare arrangements for participants who suffer an adverse event as a result of participating in the study may include advice from clinical members of the study team or the patient's treating diabetes team, or use of emergency health services.

If an adverse event occurs, there are no special compensation arrangements unless this was due to the negligence of one of the clinical investigators or due to harm resulting from study protocol design. In this case participants may have grounds for legal action for compensation. The normal national complaints mechanism will be available. In addition, any harm arising due to study design (both negligent and non-negligent) will be covered under Sponsor's insurance policy as applicable.

10.3. Risks and anticipated adverse events and adverse device effects

Known risks represent hazardous situations which may result in anticipated adverse events. In the following text, where appropriate, the term "risk" and "anticipated adverse events" are used interchangeably without affecting meaning.

10.3.1. Hypoglycaemia and hyperglycaemia

Participants with type 2 diabetes requiring insulin therapy have a pre-existing risk for hypoglycaemia and hyperglycaemia. Potential risks are:

- Risk of mild to moderate hypoglycaemia and associated symptoms such as sweating, trembling, difficulty thinking and dizziness. There is also a rare risk of severe hypoglycaemia when conscious level is altered, needing help from a third party to correct the hypoglycaemia. These risks are pre-existent in any patient with type 2 diabetes requiring insulin and the study objective is to develop systems to minimise these risks
- Risk of possible mild to moderate hyperglycaemia similar to the risk that a person with type 2 diabetes requiring insulin experiences on a daily basis

10.3.2. Blood sampling

Participants will have maximum of three blood tests during the study. Potential risks include:

- Slight discomfort or bruising at the site (common)
- Haematoma – accumulation of blood within the tissues that clots to form a solid swelling (unlikely)
- Localised infection – an infection in the tissue around the site (rare)

10.3.3. Insulin injection therapy

Potential risks associated with insulin injection therapy include:

- Slight discomfort at the time of insulin injection (common)
- Slight bruising at the site of injection (common)
- Bleeding at injection site (rare)
- Infection at the site of injection (rare)
- Insulin pen malfunction and mechanical problems (rare)
- Allergy to insulin (very rare)
- Lipodystrophy / lipoatrophy (very rare)

10.3.4. Insulin pump therapy

Potential risks and adverse device effects associated with insulin pump therapy include:

- Slight discomfort at the time of insertion of the insulin delivery cannula (common)
- Slight bruising at the site of insertion (common)
- Bleeding at insertion site (rare)
- Infection at the site of insertion (rare)
- Allergy to the insulin delivery cannula or adhesive (rare)
- Infusion set and cannula occlusions (rare)
- Insulin pump malfunction and mechanical problems (rare)
- Allergy to insulin (very rare)
- Lipodystrophy / lipoatrophy (very rare)

10.3.5. Continuous glucose monitoring

Potential risks and adverse device effects associated with CGM:

- Slight discomfort at the time of insertion of CGM (common)
- Slight bruising at the site of insertion (unlikely)
- Bleeding at insertion site (rare)
- Infection at the site of insertion (rare)
- Allergic reaction to the CGM sensor material (rare)
- Intermittent CGM failure (common)

If a skin reaction is classified as severe (the observation is noticeable and bothersome to participant and may indicate infection or risk of infection or potentially life-threatening allergic reaction), an adverse event form will be completed.

10.3.6. Retinopathy

People with type 2 diabetes have a pre-existing risk of developing diabetic retinopathy, and this risk is increased in those with sub-optimal glycaemic control. However, a rapid improvement in blood glucose levels, as may be seen with a closed-loop system, can sometimes also lead to a worsening of diabetic retinopathy in the short term. Participants will be warned of this risk, and advised to regularly attend retinopathy screening, as is part of routine clinical care.

10.3.7. Questionnaires and interviews (participants)

Participants will complete questionnaires which include questions about their expectations, private attitudes, feelings and behaviours related to diabetes and its management. It is possible that some people may find these questions to be mildly upsetting. Similar questionnaires have been used in previous research and these reactions are uncommon. A subset of participants randomised to the

closed-loop group will take part in semi-structured interviews towards the end of their participation in the trial. If a participant gets upset, the interviewer will offer to stop the interview or allow someone to skip questions if they don't want to answer them. If questionnaire or conversational responses indicate serious psychological distress as judged by the investigators, participants will be directed towards appropriate clinical service. Any treatment will be documented in the case-report form.

10.3.8. Healthcare Professional Interviews (UK only)

Healthcare professionals will be offered an interview by telephone or an online platform at a time of their choosing. They will be given opportunities to stop the interview, or not answer questions, if they wish to do so. These interviews are highly unlikely to cause upset, so the risks of taking part should be very minimal.

10.3.9. Risk analysis and residual risk associated with the investigational device

After in-depth analysis and consideration of all the potential hazards in relation to use of the CamAPS HX system in the home environment, it is concluded that the CamAPS HX system is safe, if used as intended.

Risk Assessment of the CamAPS HX system has been carried out in accordance with ISO 14971:2019. A preliminary Hazard Determination has been carried out including consideration of the questions in Annex C of ISO 14971:2019.

As per the risk management process, further risk analysis shall be undertaken post production and release as to ensure any issues raised are acted upon to ensure the CamAPS HX system continues to improve and develop.

10.4. Benefits

It is expected that the fully closed loop system may have an important role in the management of diabetes in people with type 2 diabetes.

The closed-loop system may impact on the frequency of hypoglycaemia with suspected fewer low glucose levels with closed-loop insulin delivery compared with usual care. In addition to this, higher blood glucose levels above target should be reduced with use of the closed-loop algorithm. In the closed-loop arm, participants will not need to self-administer insulin injections, which may facilitate diabetes management. In the control arm, participants will not need to monitor glucose levels with

finger-stick glucose checks, which may facilitate diabetes management. Therefore, participation in this study is likely to be beneficial for study participants.

It is possible that participants will not directly benefit from being a part of this study. However, during the run-in and training, the participant will have close contact with the study team and / or diabetes educator. The CGM data provided during the study may also help participants to optimise their usual insulin therapy and diabetes self-management.

10.5. Burdens

The study involves up to 5 visits and 6 telephone / email contacts and taking 3 blood samples. The study includes a 1-2 hour training visit on study devices and a 26 week home study period where participants will wear the study devices.

10.6. Data Safety Monitoring Board (DSMB)

An independent Data Safety Monitoring Board (DSMB) will be informed of all serious adverse events and any unanticipated adverse device effects that occur during the study and will review compiled adverse event data at periodic intervals.

11. Methods and assessments

11.1. Procedures

11.1.1. Height, weight and blood pressure

Height will be measured at the baseline visit in centimetres using calibrated measuring devices. Weight will be measured at the baseline visit, 3 month visit and end of study visit in kilograms using a calibrated electronic scale. Waist hip ratio will be measured at the baseline visit, 3 month visit and end of study visit in centimetres using a stretch-resistant tape. Blood pressure will be measured at the baseline visit, 3 month visit and end of study visit in mmHg, using an approved blood pressure monitor.

11.1.2. Venepuncture

- Venous blood samples for the measurement of HbA1c levels will be taken at baseline, at 3 months and at the final study visit. HbA1c will be measured at a central laboratory using an International Federation of Clinical Chemistry and Laboratory Medicine (IFCC) aligned method. HbA1c testing will follow National Glycohemoglobin Standardization Program (NGSP) standards. Samples will be stored locally until analysis at the central laboratory.
- Venous blood samples for the measurement of fasted lipid profile, renal function (sodium, potassium, urea, serum creatinine, estimated Glomerular Filtration Rate) and liver function (ALT, AST, ALP, yGT, bilirubin and albumin) will be taken at baseline, at 3 months and at the final study visit and will be analysed locally. Blood samples will be disposed of after analysis.

11.1.3. Urine Albumin Creatinine Ratio

A urine sample for the measurement of urine albumin creatinine ratio will be taken at the baseline visit, 3 month visit and end of study visit and analysed locally. Urine samples will be disposed of after analysis.

11.1.4. Continuous subcutaneous glucose monitoring data

At least 10 days of masked CGM data will be collected before visit 2 and pre-randomisation. Post-randomisation real-time CGM data will be automatically uploaded using a secure website. Continuous glucose monitoring data during the 26 week study period will be used to assess for efficacy. This data can also be used by the clinical team to facilitate insulin dose optimisation.

11.1.5. Insulin pump and closed-loop data

During the study the insulin pump and closed-loop data will be uploaded using a secure website.

11.2. Assessment of Safety

Safety evaluation will include reports of severe hypoglycaemia events and other adverse events and serious adverse events and adverse device effects.

11.3. Human factors assessments

The human factors assessment includes both quantitative (i.e., surveys) and qualitative (i.e., interviews) data to be gathered to provide the richest, most comprehensive characterisation of the sample and their response to the closed-loop system. Healthcare professionals working on the trial and supporting closed-loop users at UK sites will also be interviewed.

11.3.1. Questionnaires

Surveys used in this trial are listed in Table 3. In total, human factor assessment should take approximately 20 minutes to complete. Surveys will be completed following recruitment, at the midpoint and at the end of study visit. All results will be evaluated at the end of the study once all participants have completed the final study visit.

Table 3 Human Factors Assessment.

Measure	***Construct Measured/Relevant Points***
Surveys	
Problem Areas in Diabetes (PAID) Survey	20-item survey measuring diabetes-related emotional distress and covers a range of negative emotional problems of patients with diabetes (4 minutes).
Hypoglycaemia Fear Survey-version II (HFS-II)	33-item questionnaire with two subscales that measure 1) behaviours to avoid hypoglycaemia and its negative consequences and 2) worries about hypoglycaemia and its negative consequences (5 minutes).
Diabetes Treatment Satisfaction Questionnaire (DTSQ)	8 item measure developed to assess patient satisfaction with diabetes treatment (3 minutes)
EQ-5D-3L	Developed to describe and value health across a wide range of disease areas. The survey consists of two pages: the EQ-5D

	descriptive system assessing 5 dimensions of health and the EQ-5D visual analogue scale (3 minutes).
WHO-5 quality of life measure	5 item measure from the World Health Organization (WHO) that assesses health related quality of life (2 mins).
Closed-loop experience questionnaire	Feedback questionnaire on closed-loop specific experience will be completed by participants been randomised to the closed-loop intervention arm (3 minutes).

11.3.2. Participant Interviews

The qualitative study will use a cross-sectional design in which approximately 30 participants in the closed-loop arm will be interviewed after they have had approximately six months experience of using the closed-loop system. Participants will be recruited from UK sites and purposively sampled to ensure diversity in terms of age, gender, diabetes duration and sociodemographic variables such as education, ethnicity and occupation. Participants will have the option to be interviewed via telephone or online platform. Interviews will be informed by topic guides which will be revised in light of emerging findings, in line with an inductive approach. Interviews will explore participants' pre-trial diabetes management practices, everyday work and family lives, and initial expectations of using closed-loop technology before capturing their perspectives and experiences of using closed-loop to consider whether, in what ways, and why, using closed-loop technology has impacted diabetes self-management and every day/wider quality-of-life.

11.3.3 Healthcare Professional Interviews (UK only)

We will invite approximately 20 healthcare professionals (2-3 from each UK site) who are involved in supporting people using a closed-loop during the trial to take part in an interview at, or near the end of their involvement in the trial, or if they stop working on it early. Participants will have the option to be interviewed via telephone or online platform. Interviews will last around 45 minutes and be informed by topic guides which will be revised in light of emerging findings, in line with an inductive approach. Interviews will explore healthcare professionals' experiences of supporting people with T2D using a fully closed-loop system during the trial and their views about the training and resourcing needed to support system use in routine clinical care.

12. Study materials and products

12.1. Insulin

During run-in and the control intervention, rapid acting insulin analogues (insulin aspart, insulin lispro, insulin glulisine or similar) or ultra-rapid insulin analogues (Faster insulin aspart (Fiasp) or ultra-rapid Lispro (Lyumjev) or similar) and / or long acting insulin analogues (insulin glargine, insulin detemir or similar) and / or mixed insulin analogues (Novomix30, Humalog Mix25, Humalog Mix50 or similar) will be delivered subcutaneously using an insulin pen injection device or insulin pump therapy in accordance with manufacturer instructions for use.

During the closed-loop intervention period, rapid (insulin aspart, insulin lispro, insulin glulisine or similar) or ultra-rapid insulin analogue (Faster-acting insulin aspart (Fiasp) or ultra-rapid Lispro insulin (Lyumjev) or similar) will be administered via an insulin pump as described below.

12.2. Insulin injections

During run-in and the control intervention, those using subcutaneous insulin injections pre-study will continue to administer insulin using CE-marked insulin pen devices as per usual clinical practice.

12.3. Insulin pump

During the closed-loop intervention, the YpsoPump insulin pump (YpsoMed) will be used.

12.4. Continuous subcutaneous glucose monitor

During run-in and both intervention arms, Freestyle Libre 3 real-time sensor (Abbott Diabetes Care, CA, USA) or an alternative compatible CE-marked CGM system will be used. The sensor will be masked during the run-in period and unmasked during the study intervention period.

12.5. Smartphone

During the closed-loop intervention arm, a compatible unlocked smartphone hosting the CamAPS HX app (see 3.6) will be used. Participants can use their personal phone if this is compatible with the app to reduce device burden.

12.6. Computer-based algorithm

The Cambridge closed loop controller has been used safely and effectively in the closed-loop studies in both children and adults with T1D and in adults with T2D (study REC Ref. 06/Q0108/350, REC

Ref. 07/H0306/116, REC Ref. 08/H0304/75, REC Ref. 08/H0308/297, REC Ref. 09/H0306/44, REC Ref. 10/H0304/87, REC Ref. 12/EE/0155, REC Ref. 12/EE/0034, REC Ref. 12/EE/0424, REC Ref. 13/EE/0120, REC Ref. 13/WM/0498, REC Ref. 13/EE/0251, REC Ref. 13/EE/0321, REC Ref. 13/EE/0018, REC Red 15/EE/0324, REC ref 16/EE/0286, REC ref 16/EE/0380 and REC Ref 17/LO/0576), REC Red 19/LO/0728.

13. Data Analysis

Analyses of study data will be conducted to address the primary and secondary objectives of the trial. All randomised participants will be included in the analysis according to intention to treat principle.

13.1. Primary Endpoint Analysis

The primary analysis will evaluate the between-group difference in HbA1c at the end of the 26 week intervention period.

The primary analysis will be performed on an intention-to-treat basis using the treatment group assigned by randomisation. Data from participants with or without protocol violation and as appropriate, data from dropouts and withdrawals will be included in the analysis.

Mean ± SD or summary statistics appropriate to the distribution will be reported for the primary endpoint by treatment intervention. The treatment interventions will be compared using a linear mixed model with the dependent variable being the primary endpoint and the independent variable being treatment allocation adjusting for baseline HbA1c. A 95% confidence interval will be reported for the difference between the interventions based on the linear mixed model. Residual values will be examined for an approximate normal distribution. If values are highly skewed, then a transformation or robust statistical method will be used instead. However, previous experience suggests that the primary endpoint will follow an approximately normal distribution.

A 5% significance level will be used to declare statistical significance for the primary comparison. A two-sided p-value will be reported. The primary analysis will be a single statistical comparison of a single outcome measure.

A centre-effect will be explored in the analyses by evaluating for interaction between centre and treatment group on the primary outcome. A per-protocol analysis restricted to participants with a minimum of 60% CGM data during control period and 60% use of closed-loop during closed-loop period will be conducted for the primary endpoint.

A detailed analysis plan will be provided separately.

Primary endpoint hypotheses

- Null Hypothesis: There is no difference in the true mean HbA1c at the end of the 26 week period between the two treatment groups.

- Alternative Hypothesis: There is a nonzero difference in the true mean HbA1c at the end of the 26 week period between the two treatment groups.

13.2. Key and secondary endpoint analysis

For the following key endpoints, the familywise type I error rate (FWER) will be controlled at two sided α = 0.05. A gatekeeping strategy will be used, where the primary endpoint will be tested first, if passing the significance testing, other key endpoints will be tested in the order listed below using the fixed-sequence method at α = 0.05.

Key endpoints

- Proportion of time spent in the target glucose range (3.9 to 10.0mmol/l)
- Mean sensor glucose
- Proportion of time spent above target glucose (>10.0mmol/l)
- Non-inferiority for time spent below target glucose (<3.9mmol/L)

The following endpoints will be considered exploratory and Benjamini-Hochberg false discovery rate (FDR) adjusted p-values will be calculated within each subcategory below:

Secondary endpoints

Glucose related indices:

- Standard deviation and coefficient of variation of glucose
- Proportion of time with glucose <3.5mmol/l and <3.0mmol/l
- Proportion of time with glucose >13.9 mmol/l, >16.7mmol/l and >20.0mmol/l
- Binary metrics for HbA1c (HbA1c <7.0%, HbA1c <7.5%)

Insulin and other endpoints:

- Total daily insulin dose
- Body weight, waist hip ratio and Body Mass Index
- Blood pressure
- Fasted lipid profile
- Renal function as measured by sodium, potassium, urea, serum creatinine, estimated Glomerular Filtration Rate and urinary albumin creatinine ratio
- Liver function as measured by liver markers (ALT, AST, ALP, yGT, bilirubin and albumin) and FIB4 index

Trends in CGM and insulin data collected within intervention arms will be evaluated on a monthly basis and daytime (0600 to 2359) and overnight (0000 to 0559) glucose control will be evaluated separately.

For the pre-specified key endpoints and secondary endpoints, mean ± SD or summary statistics appropriate to the distribution will be reported for the primary endpoint over the 26 week period by treatment intervention and tabulated by treatment group. Analysis of secondary endpoints will parallel the primary analysis. A transformation will be applied to all highly skewed secondary endpoints.

13.3. Safety Evaluation

Safety data including severe hypoglycaemia events will be tabulated for all participants, including drop-outs and withdrawals, irrespective of whether CGM data are available and irrespective of whether closed-loop was operational. All adverse events will be listed for the entire study duration.

For each of the following safety outcomes, mean ± SD or summary statistics appropriate to the distribution will be tabulated by treatment group:

- Number of severe hypoglycaemia events
- Number of participants with any severe hypoglycaemia event
- Number of diabetic ketoacidosis events
- Number of participants with any diabetic ketoacidosis event
- Number of adverse events per participant
- Number of serious adverse events per participant

For severe hypoglycaemia (if enough events), the event rates will be compared using a repeated measures regression model.

13.4. Utility Evaluation

The amount of CGM use will be tabulated for each treatment arm, in addition to the amount of closed-loop system use in the closed-loop arm. Summary statistics appropriate to the distribution and range will be reported.

13.5. Questionnaires

Descriptive tabulations of questionnaires will be carried out, and scores will be calculated using provided scaling and scoring tools as appropriate.

13.6. Participant Interviews

To maximize rigor, at least two experienced qualitative researchers will be involved in data analysis. A thematic analysis will be undertaken by these individuals who will independently review data and write separate reports before attending regular meetings to compare their interpretations and reach agreement on recurrent themes and findings. Interviews will be read through repeatedly and cross-compared in order to identify issues and themes which cut across different people's accounts. A key aspect of the analysis will focus on understanding the impact of using a closed loop on diabetes self-management practices and quality of life compared with their accounts of self-managing their diabetes using standard insulin therapy pre-trial. A final coding frame, reflecting the initial research questions and emergent themes, will be developed once all data have been reviewed and consensus reached on key themes and findings.

13.7. Healthcare Professional Interviews

To maximise rigor, at least two experienced qualitative researchers will be involved in data analysis. These researchers will independently review interview transcripts (data immersion) and use the method of cross comparison to identify issues and themes that cut across healthcare professionals' accounts. A final coding frame, reflecting the initial research questions and emergent themes, will be developed once all data have been reviewed and consensus reached on key themes and findings.

13.8. Evaluative periods

The primary and secondary measures will be calculated from day 1 until the end of the 26 week study intervention. Where appropriate, sensor-based measures will also be calculated for day and night-time periods. The interval from 0600 to 2359 defines day-time period, 0000 to 0559 defines the night-time period.

13.9. Interim monitoring and analysis

No formal interim analysis will be performed. Interim analyses of the safety data primarily will be performed at regular intervals (at least annually) for review by the DSMB. The DSMB members will comply with DSMB charter. The DSMB will be asked to give advice on whether the accumulated data from the trial, together with results from other relevant trials, justifies continuing recruitment of further participants or further follow-up.

13.10. Adherence and retention

Protocol adherence will be assessed in each treatment group. Tabulations of protocol deviations and unscheduled visits will be included in the analysis. A flow chart will also be used to assess visit completion rates post treatment initiation.

13.11. Sample size and power calculations

Data from two completed studies (30, 40) were considered when preparing the power calculations for this study. A sample size of 96 participants (48 per group) was determined to have 90% power to detect a difference in mean HbA1c level between treatment groups, assuming a population difference of 0.8%, an effective SD of the 26 week values of 1.2, and a two-sided type 1 error rate of 0.05. This number was increased to 224 (112 per group) to account for dropouts (assuming a 20% dropout rate) and to increase the number of participants who will be exposed to the closed-loop system for an enhanced safety and feasibility assessment. Recruitment will target a minimum quota of 25% of participants using basal insulin and a minimum quota of 60% of participants using multiple daily insulin injections.

A treatment group difference of ≥0.5% represents a clinically meaningful change in the HbA1c distribution and is generally associated with at least a 50% greater proportion of participants experiencing improvement in HbA1c level by 10% or more, which was shown in the Diabetes Control and Complications Trial to be associated with a substantial risk reduction in diabetic microvascular complications. This is further supported by the results of the United Kingdom Prospective Diabetes Study, which showed that any improvement in HbA1c level is associated with a reduction in risk of diabetic complications.

13.12. Deviations from the statistical plan

Any deviations from the original statistical plan will be recorded and agreed by the Coordinating Investigator and by the Principal Investigators.

14. Case Report Forms

The Case Report Form (CRF) is the printed, optical, or electronic document designed to record all the protocol required information to be reported to the Coordinating Investigator for each study participant.

CRFs will be completed in accordance with ISO 14155:2020 GCP standard. Corrections to the CRF will be performed by striking through the incorrect entry and by writing the correct value next to the data that has been crossed out; each correction will be initialled and explained (if necessary) by the Investigator or the Investigator's authorised staff.

If any amendments to the protocol or other study documents are made, CRFs will be reviewed to determine if an amendment to these forms is also necessary.

15. Data Management

Confidentiality of participant data shall be observed at all times during the study. Personal details for each participant taking part in the research study and linking them to a unique identification number will be held locally on a study screening log in the Investigator Site File at each of the investigation centres. These details will not be revealed at any other stage during the study, and all results will remain anonymous.

Case report forms (CRFs) will be used for recording anonymised study data. The study identification number will be used on the CRF. Names and addresses will not be used.

The study identification number will be used on the blood samples that are collected and stored throughout the study. Collected samples will be stored securely and locked away. Only researchers directly involved in the study will have access to the samples.

Electronic data will be stored on password-protected computers. All paper records will be kept in locked filing cabinets, in a secure office at each of the investigation centres. Only members of the research team and collaborating institutions will have password access to the anonymised electronic data. Only members of the research teams will have access to the filing cabinet. All data will be stored in line with the General Data Protection Regulation (GDPR) (EU) 2016/679 and the Data Protection Act (DPA) 2018 and will be archived securely according to the Sponsor's archiving policy. In case of withdrawal of participants, anonymised data that were obtained before withdrawal will be used.

Direct access to the source data will be provided for monitoring, audits, REC review and regulatory authority inspections during and after the study. The fully anonymised data may be shared with third parties (EU or non-EU based) for the purposes of advancing management and treatment of diabetes.

Appropriate procedures agreed by the Coordinating Investigator and Clinical Principal Investigators will be put in place for data review, database cleaning and issuing and resolving data queries.

15.1. Switzerland

Study data will be collected, managed and stored using REDCap electronic data capture tools hosted at the Department of Anaesthesiology and Pain Medicine, Inselspital, Bern University Hospital. REDCap® (Research Electronic Data Capture) is a secure application designed to support data capture for research studies, providing 1) an intuitive interface for validated data entry; 2) audit trails for tracking data manipulation and export procedures; 3) automated export procedures for seamless data downloads to common statistical packages; and 4) procedures for importing data from external sources [https://projectredcap.org/resources/citations/].

The study team in Bern will use the identical eCRF on REDCap.

Data within REDCap® are stored in relational database engines like PostgreSQL or Microsoft SQL server. REDCap® provides web application security and can be configured for Secure Socket Layer (SSL) encrypted data transfer if needed.

15.1.1 Data security, access and back-up

The study database in REDCap® can only be accessed by designated investigator staff entering a user name and password. The application has a group and role-based security model. Each user belongs to one or more security groups with specific sets of permissions about folder or projects in the system. Only dedicated site administrators have access to the admin console, enabling user management and changing security settings.

All events are recorded in the user event list of the audit log files. Data are stored and visualised in data grids either in the format of datasets, lists or assays. Each change of data is tracked and documented in corresponding audit log files.

The servers are behind a firewall and cannot be accessed through the internet. They are located in locked dedicated server rooms with restricted access. Apache HTTP Server and REDCap® were configured to run under Secure Sockets Layer (SSL) which implies that data is encrypted and transmitted securely.

Available disk space is monitored actively. If free disk space is less than 10%, administrators get an email, and more storage capacity will be added accordingly.

All servers are regularly backed up on storage servers in a separate server room using a multi-level system.

15.1.2 Analysis and archiving

REDCap® enables data analysis by integrated tools (e.g. creation of reports, charts and figures). The data export function allows exporting data in vaious statistical formats (Microsoft Excel, CSV, PDF, SAS, Stata, R, SPSS), with the option to select all data or partial data, suitable for transfer to a statistical software package of choice.

15.1.3 Electronic and central data validation

An automatic validation program within RedCap® will check for data discrepancies and, by generating appropriate error messages, allow modification or verification of the entered data by the investigator staff.

15.2. France

Study data will be collected, managed and stored using a data management system hosted by University Hospital Toulouse.

15.3. Germany

Study data will be collected, managed and stored using REDCap electronic data capture tools hosted at the for Medicine II at University Hospital of Freiburg.
The study team in Freiburg will use the identical eCRF on REDCap.

15.4. Czech Republic

Study data will be collected, managed and stored using hospital patient data management system hosted by Institute for Clinical and Experimental Medicine (IKEM), Prague, Czech Republic (Zlatokop system). This system has all security features to handle data from all patients treated in IKEM. It also has a special module designed for clinical trials with an interface for validated data entry, audit trails for tracking data manipulation and export procedures, ability to export data to common statistical packages and ability to import data from external sources.

15.5. Australia

The REDCap (Research Electronic Data Capture) study database will be used to collect, manage and store study data hosted on centralised servers through the Department of Medicine, The University of Melbourne, Australia.

All study data will be entered into the relevant electronic case report form (eCRF) in the trial database.

15.5.1 Data security, access and back-up

The study database in REDCap® can only be accessed by HREC approved study personnel by entering a study specific user name and password.

All events are recorded in the user event list of the audit log files. Data are stored and visualised in data grids either in the format of datasets, lists or assays. Each change of data is tracked and documented in corresponding audit log files.

All servers are backed up daily on storage servers in a separate server room using a multi-level system.

16. Ethics

The study will be conducted in accordance with the Declaration of Helsinki Ethical Principles for Medical Research involving Human Subjects (64th WMA General Assembly, Fortaleza, Brazil, October 2013).

16.1. Research Ethics Committee and Institutional Review Board

Prior to commencement of the study, the protocol, any amendments, participant information and informed consent forms, any other written information to be provided to the participant, participant recruitment procedures, current investigator CVs, and any other documents as required by the Research Ethics Committee (REC) or Institutional Review Board will be submitted. Written approval will be obtained from the REC prior to the commencement of the study. Any additional requirements imposed by the REC or regulatory authority shall be followed.

16.2. Informed consent of study participants

In obtaining and documenting informed consent, the investigator will comply with the applicable regulatory requirements and will adhere to GCP standards and to the ethical principles that have their origin in the Declaration of Helsinki. Prior to the start of the study, the Investigator will obtain

favourable ethical opinion of the written informed consent form and any other written information to be provided to participants.

Participants will be given full verbal and written information regarding the objectives and procedures of the study and the possible risks involved. Participants will be given ample time to consider participation in the study. All participants will be informed of their right to leave the study at any time, without stating any reason, and without any negative consequences to their subsequent medical treatment. The participant will be informed in a timely manner should any new information become available during the course of the study that may affect their well-being, safety and willingness to participate in the study.

Written consent will be obtained from participants according to REC requirements. The signed informed consent forms will be photocopied, originals filed in the Investigator's Site File, a copy placed in the patient's notes and a copy given to the participant.

17. Amendments to the protocol

Any substantial amendments to the protocol and other documents shall be notified to, and approved by, the Research Ethics Committee or Institutional Review Board prior to implementation as per nationally agreed guidelines.

18. Deviations from the protocol

Deviations from the protocol should not occur without prior approval of the REC or sponsor except under emergency circumstances, to protect the rights, safety and well-being of participants. If deviations do occur, they will be documented, stating the reason and the date, the action taken, and the impact for the participant and for the study. The documentation will be kept in the Investigator's Site File.

Deviations affecting the participant's rights, safety and well-being or the scientific integrity of the study will be reported to the REC and sponsor as soon as possible in a timely manner, following nationally-agreed guidelines.

19. Study management

19.1. Data and Safety Monitoring Board (DSMB)

An independent Data Safety Monitoring Board (DSMB) will comprise an independent chairperson and two external experts. The DSMB aims to safeguard the interests of trial participants, assess the safety data, critical clinical performance and effectiveness endpoints of the interventions during the trial, and monitor the overall conduct of the clinical trial. Furthermore, the DSMB will recommend to the sponsor whether to continue, suspend, modify, or stop the clinical investigation (ISO 14155: 3.17).

The DSMB should receive and review the progress and accruing data of the project and provide advice on the conduct of the trial. The DSMB will be informed of all serious adverse events and any unanticipated adverse device effects that occur during the study and will review compiled adverse event data at periodic intervals.

20. Responsibilities

20.1. Coordinating Investigator

The Coordinating Investigator (CI) is the person with overall responsibility for the research in a multicentre clinical investigation. All ethical applications will be submitted by the CI. The CI is accountable for the conduct of the study and will ensure that all study personnel are adequately qualified and informed about the protocol, any amendments to the protocol, the study treatments and procedures and their study related duties. The CI should maintain a list of appropriately qualified persons to whom he/she has delegated specified significant study-related duties.

20.2. Principal Investigator

The principal investigator is responsible for conducting the clinical investigation at an investigation site.

If a clinical investigation is conducted by a team of individuals at an investigation site, the Principal Investigator is responsible for leading the team.

Whether this is the responsibility of an individual or an institution can depend on national regulations. The PI should maintain a list of appropriately qualified persons to whom he/she has delegated specified significant study-related duties.

21. Reports and Publications

Data will be submitted for publication in internationally peer-reviewed scientific journals; members of the investigator group will all be co-authors. The privacy of each participant and confidentiality of their information shall be preserved in reports and publication of data.

22. Timetable

Inclusion of the first participant in the study is planned to take place in September 2024. The end of the study will be defined as the date of the final visit of the last participant. Expected completion of the last participant is March 2027 and the planned completion of the Clinical Study Report is July 2027.

23. Retention of Study Documentation

Participant notes must be kept for the maximum time period as permitted by each relevant institution. Other source documents (inc. sponsor file) and the Investigator's Site File must be retained for at least 15 years, in line with MDR (annex XV, chapter III). The Principal Investigator will archive the documentation pertaining to the study after completion or discontinuation of the study.

23.1 Switzerland

The Sponsor and Principal Investigator will ensure that all data is archived and secured in the database for a minimum of 10 years after completion or discontinuation of the trial. Access will be restricted to research team members.

23.2 France

The Sponsor and Principal Investigator will ensure that all data is archived and secured in the database for a minimum of 15 years after completion or discontinuation of the trial. Access will be restricted to research team members.

23.3 Germany

The Sponsor and Principal Investigator will ensure that all data is archived and secured in the database for a minimum of 10 years after completion or discontinuation of the trial.

23.4 Austria

Study specific documents are archived for 10 years according to MDR Annex XV, Chapter III, Art. 1 and 3.

23.5 Czech Republic

The Investigator will retain the study documents for at least 10 years after the completion or discontinuation of the clinical trial.

23.6 Australia

All source data, clinical records, and laboratory data relating to the study will be archived within the Diabetes Technology Research Group at St Vincent's Hospital Melbourne, under the supervision of the Chief investigator for a minimum of 15 years after the completion of the study.

24. Indemnity statements

The principal investigators are indemnified to cover negligent harm to patients participating in the study by their membership of medical defence organisations. Indemnity for any harm arising from the conduct of research will be provided according to local arrangements in respective centre.

24.1. UK

National Health Service indemnity cover will apply for any claims arising from management and conduct of research. Any liability arising from study design will be covered by the clinical trial insurance policy organised by the University of Cambridge.

24.2 Switzerland

The trial qualifies for a category A1 performance study and is therefore exempt from liability coverage requirements (ClinO Art 12).

24.3 France

Study insurance will be provided by the University Hospital Toulouse, France. A copy of the certificate will be transmitted to the French Ethics Committee and competent authority if necessary.

24.4 Germany

Study insurance will be provided by the ECCLESIA mildenberger HOSPITAL GmbH. A copy of the certificate will be filed in the investigator site file and the trial master file.

24.5 Austria

Study insurance will be provided by the Medical University of Graz. A copy of the certificate will be filed in the investigator site file and the trial master file.

24.6 Czech Republic

A standard clinical trial insurance will be provided for all study participants by the Sponsor.

24.7 Australia

St Vincent's Hospital Melbourne will provide indemnity cover will apply for any claims arising from management and conduct of research. A copy of the certificate will be filed in the investigator site file and the trial master file.

25. References

1. Association AD. Diagnosis and classification of diabetes mellitus. Diabetes Care. 2010;33 Suppl 1:S62-9.

2. Marín-Peñalver JJ, Martín-Timón I, Sevillano-Collantes C, Del Cañizo-Gómez FJ. Update on the treatment of type 2 diabetes mellitus. World J Diabetes. 2016;7(17):354-95.

3. Lascar N, Brown J, Pattison H, Barnett AH, Bailey CJ, Bellary S. Type 2 diabetes in adolescents and young adults. Lancet Diabetes Endocrinol. 2018;6(1):69-80.

4. Federation ID. IDF Diabetes Atlas, 9th edn. Brussels, Belgium: 2019. Available at: https://www.diabetesatlas.org.

5. Skyler JS, Bergenstal R, Bonow RO, Buse J, Deedwania P, Gale EA, et al. Intensive glycemic control and the prevention of cardiovascular events: implications of the ACCORD, ADVANCE, and VA diabetes trials: a position statement of the American Diabetes Association and a scientific statement of the American College of Cardiology Foundation and the American Heart Association. Diabetes Care. 2009;32(1):187-92.

6. Orchard TJ, Temprosa M, Barrett-Connor E, Fowler SE, Goldberg RB, Mather KJ, et al. Long-term effects of the Diabetes Prevention Program interventions on cardiovascular risk factors: a report from the DPP Outcomes Study. Diabet Med. 2013;30(1):46-55.

7. Cahn A, Miccoli R, Dardano A, Del Prato S. New forms of insulin and insulin therapies for the treatment of type 2 diabetes. Lancet Diabetes Endocrinol. 2015;3(8):638-52.

8. Wright A, Burden AC, Paisey RB, Cull CA, Holman RR, Group UKPDS. Sulfonylurea inadequacy: efficacy of addition of insulin over 6 years in patients with type 2 diabetes in the U.K. Prospective Diabetes Study (UKPDS 57). Diabetes Care. 2002;25(2):330-6.

9. Rys P, Wojciechowski P, Rogoz-Sitek A, Niesyczyński G, Lis J, Syta A, et al. Systematic review and meta-analysis of randomized clinical trials comparing efficacy and safety outcomes of insulin glargine with NPH insulin, premixed insulin preparations or with insulin detemir in type 2 diabetes mellitus. Acta Diabetol. 2015;52(4):649-62.

10. NICE guideline [NG 28] Type 2 diabetes in adults: management.: National Institute for Health and Care Excellence; 2015 Dec 2015.

11. Holman RR, Paul SK, Bethel MA, Matthews DR, Neil HA. 10-year follow-up of intensive glucose control in type 2 diabetes. The New England journal of medicine. 2008;359(15):1577-89.

12. Edridge CL, Dunkley AJ, Bodicoat DH, Rose TC, Gray LJ, Davies MJ, et al. Prevalence and Incidence of Hypoglycaemia in 532,542 People with Type 2 Diabetes on Oral Therapies and Insulin: A Systematic Review and Meta-Analysis of Population Based Studies. PLoS One. 2015;10(6):e0126427.

13. Phillip M, Danne T, Shalitin S, Buckingham B, Laffel L, Tamborlane W, et al. Use of continuous glucose monitoring in children and adolescents (*). Pediatr Diabetes. 2012;13(3):215-28.

14. Kordonouri O, Hartmann R, Pankowska E, Rami B, Kapellen T, Coutant R, et al. Sensor augmented pump therapy from onset of type 1 diabetes: late follow-up results of the Pediatric Onset Study. Pediatr Diabetes. 2012;13(7):515-8.

15. Bergenstal RM, Klonoff DC, Garg SK, Bode BW, Meredith M, Slover RH, et al. Threshold-based insulin-pump interruption for reduction of hypoglycemia. N Engl J Med. 2013;369(3):224-32.

16. Ly TT, Nicholas JA, Retterath A, Lim EM, Davis EA, Jones TW. Effect of sensor-augmented insulin pump therapy and automated insulin suspension vs standard insulin pump therapy on hypoglycemia in patients with type 1 diabetes: a randomized clinical trial. JAMA. 2013;310(12):1240-7.

17. Hovorka R. Closed-loop insulin delivery: from bench to clinical practice. Nature reviews Endocrinology. 2011;7(7):385-95.

18. Hovorka R, Allen JM, Elleri D, Chassin LJ, Harris J, Xing D, et al. Manual closed-loop insulin delivery in children and adolescents with type 1 diabetes: a phase 2 randomised crossover trial. Lancet. 2010;375(9716):743-51.

19. Elleri D, Allen JM, Kumareswaran K, Leelarathna L, Nodale M, Caldwell K, et al. Closed-loop basal insulin delivery over 36 hours in adolescents with type 1 diabetes: randomized clinical trial. Diabetes care. 2013;36(4):838-44.

20. Nimri R, Danne T, Kordonouri O, Atlas E, Bratina N, Biester T, et al. The "Glucositter" overnight automated closed loop system for type 1 diabetes: a randomized crossover trial. Pediatric diabetes. 2013;14(3):159-67.

21. Hovorka R, Elleri D, Thabit H, Allen JM, Leelarathna L, El-Khairi R, et al. Overnight closed loop insulin delivery in young people with type 1 diabetes: A free-living randomised clinical trial. Diabetes Care. 2014;37(5):1204-11.

22. Thabit H, Tauschmann M, Allen JM, Leelarathna L, Hartnell S, Wilinska ME, et al. Home Use of an Artificial Beta Cell in Type 1 Diabetes. N Engl J Med. 2015;373(22):2129-40.

23. Nimri R, Muller I, Atlas E, Miller S, Kordonouri O, Bratina N, et al. Night glucose control with MD-Logic artificial pancreas in home setting: a single blind, randomized crossover trial-interim analysis. Pediatric diabetes. 2014;15(2):91-9.

24. Tauschmann M, Allen JM, Wilinska ME, Thabit H, Stewart Z, Cheng P, et al. Day-and-Night Hybrid Closed-Loop Insulin Delivery in Adolescents With Type 1 Diabetes: A Free-Living, Randomized Clinical Trial. Diabetes Care. 2016;Jan 6 [Epub ahead of print].

25. Leelarathna L, Dellweg S, Mader JK, Allen JM, Benesch C, Doll W, et al. Day and night home closed-loop insulin delivery in adults with type 1 diabetes: three-center randomized crossover study. Diabetes Care. 2014;37(7):1931-7.

26. Thabit H, Lubina-Solomon A, Stadler M, Leelarathna L, Walkinshaw E, Pernet A, et al. Home use of closed-loop insulin delivery for overnight glucose control in adults with type 1 diabetes: a 4-week, multicentre, randomised crossover study. Lancet Diabetes Endocrinol. 2014;2(9):701-9.

27. Thabit H, Hartnell S, Allen JM, Lake A, Wilinska ME, Ruan Y, et al. Closed-loop insulin delivery in inpatients with type 2 diabetes: a randomised, parallel-group trial. The Lancet Diabetes & Endocrinology. 2017;5(2):117-24.

28. Bally L, Thabit H, Hartnell S, Andereggen E, Ruan Y, Wilinska ME, et al. Closed-loop insulin delivery for glycemic control in noncritical care. New England Journal of Medicine. 2018;379(6):547-56.

29. Boughton CK, Bally L, Martignoni F, Hartnell S, Herzig D, Vogt A, et al. Fully closed-loop insulin delivery in inpatients receiving nutritional support: a two-centre, open-label, randomised controlled trial. The Lancet Diabetes & Endocrinology. 2019;7(5):368-77.

30. Daly AB, Boughton CK, Nwokolo M, Hartnell S, Wilinska ME, Cezar A, et al. Fully automated closed-loop insulin delivery in adults with type 2 diabetes: an open-label, single-center, randomized crossover trial. Nature Medicine. 2023.

31. Boughton CK, Tripyla A, Hartnell S, Daly A, Herzig D, Wilinska ME, et al. Fully automated closed-loop glucose control compared with standard insulin therapy in adults with type 2 diabetes requiring dialysis: an open-label, randomized crossover trial. Nature Medicine. 2021;27(8):1471-6.

32. Elleri D, Allen JM, Tauschmann M, El-Khairi R, Benitez-Aguirre P, Acerini CL, et al. Feasibility of overnight closed-loop therapy in young children with type 1 diabetes aged 3-6 years: comparison between diluted and standard insulin strength. BMJ Open Diabetes Res Care. 2014;2(1):e000040.

33. Thabit H, Tauschmann M, Allen JM, Leelarathna L, Hartnell S, Wilinska ME, et al. Home use of an artificial beta cell in type 1 diabetes. N Engl J Med. 2015;373(22):2129-40.

34. Tauschmann M, Allen JM, Wilinska ME, Thabit H, Stewart Z, Cheng P, et al. Day-and-Night Hybrid Closed-Loop Insulin Delivery in Adolescents With Type 1 Diabetes: A Free-Living, Randomized Clinical Trial. Diabetes care. 2016;39(7):1168-74.

35. Stewart ZA, Wilinska ME, Hartnell S, Temple RC, Rayman G, Stanley KP, et al. Closed-Loop Insulin Delivery during Pregnancy in Women with Type 1 Diabetes. The New England journal of medicine. 2016;375(7):644-54.

36. Hovorka R, Canonico V, Chassin LJ, Haueter U, Massi-Benedetti M, Orsini Federici M, et al. Nonlinear model predictive control of glucose concentration in subjects with type 1 diabetes. Physiol Meas. 2004;25(4):905-20.

37. Bally L, Thabit H, Kojzar H, Mader JK, Qerimi-Hyseni J, Hartnell S, et al. Day-and-night glycaemic control with closed-loop insulin delivery versus conventional insulin pump

therapy in free-living adults with well controlled type 1 diabetes: an open-label, randomised, crossover study. The lancet Diabetes & endocrinology. 2017;5(4):261-70.

38. Kumareswaran K, Thabit H, Leelarathna L, Caldwell K, Elleri D, Allen JM, et al. Feasibility of closed-loop insulin delivery in type 2 diabetes: a randomized controlled study. Diabetes care. 2014;37(5):1198-203.

39. Thabit H, Hartnell S, Allen JM, Lake A, Wilinska ME, Ruan Y, et al. Closed-loop insulin delivery in inpatients with type 2 diabetes: a randomised, parallel-group trial. Lancet Diabetes Endocrinol. 2016.

40. Martens T, Beck RW, Bailey R, Ruedy KJ, Calhoun P, Peters AL, et al. Effect of Continuous Glucose Monitoring on Glycemic Control in Patients With Type 2 Diabetes Treated With Basal Insulin: A Randomized Clinical Trial. (1538-3598 (Electronic)).

Regulatory References:

1. Declaration of Helsinki, Version October 2013

2. Medical Device Regulation (EU) 2017/745 of 5 April 2017 (MDR)

3. MDCG 2020-10/1 Safety reporting in clinical investigations of medical devices under the Regulation (EU) 2017/745

4. EN ISO 14155:2020: Clinical investigation of medical devices for human subjects - Good clinical practice

5. Swiss Ordinance on Clinical Trials with Medical Devices (ClinO-MD), of 1 July 2020

26. Document amendment history

Version number	Date	Amendment information
3.0	06.11.2024	1. **Addition of new Principal Investigator and Study Site** Dr. Emma Wilmot was added as a new Principal Investigator, and the University Hospitals of Derby and Burton NHS Foundation Trust was included as an additional study site to ensure recruitment targets are achieved.<br>2. **Correction of Typographical Error in Study Synopsis** A typographical error was corrected in the study synopsis: 'Talking' a medical history has been updated to 'taking' a medical history.<br>3. **Minor clarification to Figure 1** For clarity, the term 'android' has been removed from the protocol.<br>4. **Clarification of Recruitment and Informed Consent section** Section 6.2. has been clarified to specify that local GP surgeries within the region of the study site will serve as Participant Identification Centres (PICs) for the UK study sites.<br>5. **The protocol version number and date have been updated.**
4.0	08.01.2025	1. **Addition of Healthcare Professional Interviews** Healthcare professionals working on the trial and supporting closed-loop users at UK sites will be interviewed towards the end of their involvement in the trial.<br>2. **Modification of participant interviews design** Instead of using a longitudinal design, the qualitative interview study will use a cross-sectional design in which selected participants in the closed-loop arm will be interviewed once after they have had approximately six months experience of using the closed-loop system.<br>3. **Clarification of child-bearing age**<br>4. **Clarification of inclusion of participants with diabetes retinal disease**

5.0	21.05.2025	1. **Addition of new Principal Investigators (PIs) and Study Sites** Prof David O'Neal was added as a new PI, and St Vincent's Hospital Melbourne, Victoria, Australia was included as an additional study site in Australia. Prof. Dr. Norbert Hermanns and Prof. Dr. Bernhard Kulzer were added as new PIs, and Forschungsinstitut Diabetes-Akademie Bad Mergentheim (FIDAM GmbH) was included as an additional study site in Germany. New PIs and study sites were added to ensure recruitment targets are achieved.<br><br>2. **Addition of local requirements specific to Australia** (i) Exclusion criteria, (ii) arrangement in reporting of serious adverse events and serious adverse device effects, (iii) data management, (iv) arrangement in retention of study documentation, and (v) indemnity statements were added to meet the site-specific and local requirements in Australia.<br><br>3. **Change of personnel for study coordinator** Angel Tseung has been added as Study coordinator.
6.0	14.10.2025	1. **Addition of co-Investigator** Prof Cuong Nguyen Dang and Dr Vilashini Arul Devah were added as co-Investigators at Manchester University NHS Foundation Trust to ensure recruitment targets are achieved. The text 'Manchester Royal Infirmary' was removed to enable coverage of participating hospitals under Manchester University NHS Foundation Trust.<br><br>2. **Addition of missing text in the list of participating sites** Forschungsinstitut Diabetes-Akademie Bad Mergentheim (FIDAM GmbH), Bad Mergentheim, Germany was added as participating site in Protocol v5.0 dated 21.05.2025. Missing text was added to include the organisation to the list of participating sites.<br><br>3. **Removal of duplicated exclusion criteria** Duplicated text on 'Medically documented allergy towards the adhesive' was removed whereas 'Medically documented allergy towards the adhesive (glue) of plasters' remained in exclusion criteria.
