# Supplementary material for: Assessing the efficacy, safety and utility of fully closed-loop insulin delivery compared to standard insulin therapy with a continuous glucose monitor in adults with type 2 diabetes (COYOTE study): a randomised parallel study protocol
Source: BMJ Open. 2026 May 19;16(5):e115464. doi: 10.1136/bmjopen-2025-115464 (PMC13202074; doi:10.1136/bmjopen-2025-115464)
Supplement: Supplementary file 2 [file bmjopen-2025-115464.reviewer_comments.pdf]

PEER REVIEW HISTORY

BMJ Open publishes all reviews undertaken for accepted manuscripts. Reviewers are asked to complete a checklist review form and are provided with free text boxes to elaborate on their assessment. These free text comments are reproduced below.

ARTICLE DETAILS

Title (Provisional)

Assessing the efficacy, safety and utility of fully closed-loop insulin delivery compared to standard insulin therapy with a continuous glucose monitor in adults with type 2 diabetes (COYOTE study): a randomised parallel study protocol

Authors

Seese, Rachel; Boughton, Charlotte; Tseung, Angel; Uy, Rona; Wilinska, Malgorzata E; Thabit, Hood; Cheah, Yee S; Neupane, Sankalpa; Hussain, Sufyan; Choudhary, Pratik; Wilmot, Emma G; Bally, Lia; Hanaire, Hélène; Mader, Julia K; Martin, Haluzik; O'Neal, David Norman; Lawton, Julia; Rankin, David; Kollman, Craig; Dunseath, Gareth; Hovorka, Roman

---

VERSION 1 - REVIEW

**Reviewer**	**1**
**Name**	**Hashimoto, Yoshitaka**
**Affiliation**	**Kyoto Prefectural University of Medicine, Graduate School of Medical Science, Department of Endocrinology and Metabolism**
**Date**	**15-Feb-2026**
**COI**	**None**

Review of ”Assessing the efficacy, safety and utility of fully closed-loop insulin delivery compared to standard insulin therapy with a continuous glucose monitor in adults with type 2 diabetes (COYOTE study): a randomised parallel study protocol”

This paper is a protocol paper on the utility of fully closed-loop insulin delivery in type 2 diabetes. The reviewer requests more detailed information on the following points only.

1. Among insulin users, there is a wide range, from multiple daily injections to those using only once-daily basal insulin. Shouldn't this be considered during randomization?

2. The insulin dosage adjustment method for the standard treatment group should be described in more detail.

**Reviewer**	**2**
**Name**	**Scifres, Christina**
**Affiliation**	**Indiana University School of Medicine**
**Date**	**03-Mar-2026**
**COI**	**None**

Thank you for the opportunity to review the manuscript by Seese, et al. entitled "Assessing the efficacy, safety and utility of fully closed-loop insulin delivery compared to standard insulin therapy with a

continuous glucose monitor in adults with type 2 diabetes

(COYOTE study): a randomised parallel study protocol." This manuscript is well-written, and I have only a few comments.

1. The manuscript provides a comprehensive and clear overview of the study protocol. However, what I found missing was a discussion section at the end to summarize this work in the context of what is known about T2D care and any ongoing studies exploring similar questions.

2. This protocol paper would also benefit from a discussion of the strengths and limitations of this proposed work. Here are just a few thoughts regarding some of the potential limitations or challenges, and I suspect that the authors have considered more.

a. There may be certain study design limitations that could ultimately affect the results. For example, participants must be on either a SGLT2 Inhibitor or a GLP-1 RA prior to enrollment of have been offered these therapies in the past. Enrollment is not stratified by other diabetes therapies, which means it possible that the authors may end up with imbalance in other diabetes therapies that could influence the results.

b. Access to GLP-1 RA and diabetes technology is a challenge in many parts of the world, which could limit the generalizability of these results if the authors do find a benefit of the closed-loop insulin delivery system.

c. Exclusion of pregnant patients may be necessary for study design, but important to include this as a limitation.

VERSION 1 - AUTHOR RESPONSE

<u>Reviewer: 1</u>

Comment 1

Among insulin users, there is a wide range, from multiple daily injections to those using only once-daily basal insulin. Shouldn't this be considered during randomization?

Response: Thank you for this Comment. HbA1c is being used for randomisation stratification which we anticipate will reflect to some degree participant baseline insulin regimens. The proportion of participants using basal or more intensive therapy may differ between groups therefore this has been highlighted as a study limitation in the strengths and limitation section (Page 5).

Comment 2

The insulin dosage adjustment method for the standard treatment group should be described in more detail.

Response: Thank you for this Comment. This is a pragmatic study design to reflect current clinical practice. Beyond initial training on study devices, there is no optimisation of insulin therapy by the study team in either group. Participants and/or the clinical team are free to adjust diabetes therapy as per usual clinical practice. This has been added to the manuscript (Page 14).

<u>Reviewer: 2</u>

Comment 1

The manuscript provides a comprehensive and clear overview of the study protocol. However, what I found missing was a discussion section at the end to summarize this work in the context of what is known about T2D care and any ongoing studies exploring similar questions.

Response: Thank you for this suggestion. We have included a short summary entitled “Expected impact” to the end of the protocol (Page 21-22).

Comment 2

This protocol paper would also benefit from a discussion of the strengths and limitations of this proposed work. Here are just a few thoughts regarding some of the potential limitations or challenges, and I suspect that the authors have considered more.

Response: Thank you for this Comment. We have added a section on limitations of the study in the strength and limitations section (Page 5).

Comment 2a

There may be certain study design limitations that could ultimately affect the results. For example, participants must be on either a SGLT2 Inhibitor or a GLP-1 RA prior to enrolment of have been offered these therapies in the past. Enrolment is not stratified by other diabetes therapies, which means it possible that the authors may end up with imbalance in other diabetes therapies that could influence the results.

Response: Thank you for this Comment. The study protocol is pragmatic in design with regards inclusion criteria to support generalisability of the findings. The use of either/both of these high efficacy glucose lowering medications was to ensure that the study cohort reflects the current best practice. By including only those who have been offered these therapies it is likely that most participants will be on one or both of these medications therefore significant imbalances between groups is unlikely. It is not possible to stratify by all diabetes therapies with this sample size. We have added the lack of stratification by non-insulin glucose lowering therapies to the study limitations (Page 5).

Comment 2b

Access to GLP-1 RA and diabetes technology is a challenge in many parts of the world, which could limit the generalizability of these results if the authors do find a benefit of the closed-loop insulin delivery system.

Response: Thank you for this Comment. We agree that lack of access to advanced diabetes therapies limits generalisability. This study aims to investigate whether fully automated insulin delivery is safe and efficacious in people with T2D requiring insulin but will not be able to address issues of access to advanced therapies globally.

Comment 2c

Exclusion of pregnant patients may be necessary for study design, but important to include this as a limitation.

Response: Thank you for highlight this. We have added this as a limitation (Page 5).

VERSION 2 - REVIEW

Reviewer 1

Name Hashimoto, Yoshitaka

Affiliation Kyoto Prefectural University of Medicine, Graduate School of Medical Science, Department of Endocrinology and Metabolism

Date 17-Mar-2026

COI

This reviewer has no further comments.

Reviewer 2

Name Scifres, Christina

Affiliation Indiana University School of Medicine

Date 10-Apr-2026

COI

The authors have adequately responded to my prior comments, and I have no additional critiques.
